# Assessment of unintentional acute pesticide poisoning among smallholder vegetable farmers in Trinidad and Jamaica

**DOI:** 10.3389/fpubh.2024.1470276

**Published:** 2024-11-05

**Authors:** Dwight E. Robinson, Alexander M. Stuart, Sheila Willis, Joey P. Salmon, Jeet Ramjattan, Wayne Ganpat, Stephanie Williamson, Keith F. Tyrell, Duraisamy Saravanakumar

**Affiliations:** ^1^Department of Life Sciences, Faculty of Science and Technology, The University of West Indies, Mona, Jamaica; ^2^Pesticide Action Network UK, Brighthelm Centre, Brighton, United Kingdom; ^3^Faculty of Food and Agriculture, The University of West Indies, St. Augustine, Trinidad and Tobago

**Keywords:** acetamiprid, alpha-cypermethrin, alternatives, highly hazardous pesticides, paraquat, personal protective equipment (PPE), pesticide exposure, profenofos

## Abstract

Poisoning caused by pesticides is widely recognized as a major public health problem among smallholder farmers and rural communities, including in the Caribbean. However, a lack of quality data impedes understanding of the problem and hampers the development of effective strategies for its management. To better understand the prevalence of unintentional acute pesticide poisoning (UAPP) in Trinidad and Tobago and Jamaica and the pesticides and practices involved, we conducted a cross-sectional survey of 197 and 330 vegetable farmers in Trinidad and Jamaica, respectively. The findings from this study revealed a high incidence of self-reported health effects from occupational pesticide exposure, with 48 and 16% of respondents, respectively, experiencing symptoms of UAPP within the previous 12 months. Furthermore, the substantial proportion of UAPP incidents were associated with a few highly hazardous pesticides (HHPs), particularly lambda-cyhalothrin, acetamiprid, and profenofos in Jamaica, and alpha-cypermethrin, paraquat and lambda-cyhalothrin in Trinidad. Given the well-documented adverse effects of these chemicals on human health, the results of this study should be of significant concern to health authorities in Jamaica and Trinidad. This clearly indicates an urgent need for improved regulation and safer alternatives to the use of HHPs, as well as the promotion of alternatives. We provide policy recommendations and identify alternatives to HHPs for tropical vegetable production.

## 1 Introduction

Since the introduction of synthetic pesticides to agriculture in the 1940s, their use has increased globally year on year, reaching 3.54 million metric tons in 2021 (1). Global pesticide use has increased by 74% since 1990 (2) and by 20% between 2008 and 2018 (3). In the Carribean, farmers have been encouraged to increase local food production, particularly fresh fruits and vegetables and in response, farmers have increased their use of pesticides. Pesticides are generally applied at rates and frequencies that are inconsistent with the label instructions (4). As a result, over time, problems such as pesticide resistance and harms to the beneficial organisms that keep pest populations in check can drive escalating pesticide use and harms (5–7). Many farmers have developed an unhealthy reliance on pesticides, resulting in an increase in the importation and use of pesticides in the Caribbean (8).

The high and frequent use of pesticides in the agricultural sector undoubtedly puts farmers at an increased risk of exposure to pesticides, particularly in the dilution and application process (9) and sometimes with detrimental consequences (10, 11). Despite efforts by the Caribbean states and different stakeholders in the pesticide industry to encourage the use of personal protective equipment (PPE) by Caribbean farmers, the percentage of farmers fully and properly wearing PPE remains generally low. Studies in Jamaica and Barbados revealed that only 64.9 and 60% of farmers reported using PPE, respectively (12, 13).

Poisoning caused by pesticides is widely recognized as a major public health problem in low- and middle-income countries (LMICs) in particular (14–16). While there is a paucity of data on the occupational hazards associated with pesticide use by farmers in the Caribbean, there is evidence to suggest that acute and chronic toxicity effects of pesticides are prevalent among Caribbean farmers and rural communities (17, 18). Worldwide, it is estimated 385 million agricultural workers experience unintentional acute pesticide poisoning (UAPP) every year (19). Furthermore, self-poisoning with pesticides accounts for as many as 168,000 deaths annually (19, 20). The number of reported cases of acute pesticide poisoning ought to be a major concern, yet these estimates are likely to be significantly underestimated as studies have shown that a very high number of unintentional poisonings go unreported or misreported (21–23).

High levels of UAPP also signal a potentially larger problem of chronic pesticide exposure, which is more difficult to quantify. Epidemiological studies have established associations between occupational exposure to pesticides and chronic health impacts such as cancers, reproductive and developmental disorders and neurologial diseases, for example Parkinson’s disease (18, 24).

Women, particularly expectant and nursing mothers, are especially vulnerable to pesticide poisoning (25). Women play a significant role in agriculture, representing an estimated 43% of the agricultural labor force worldwide (26). In the Caribbean this number is lower, at around 25%. In Jamaica it is around 28% and in Trinidad and Tobago it is 17%.

Pesticide poisoning places a significant economic burden on LMICs. According to a UNEP study, the health-related expenses associated with pesticide poisoning in smallholder farming across 37 sub-Saharan countries, encompassing lost work days, outpatient medical treatment, and inpatient hospitalization, reached an estimated US$4.4 billion in 2005 (27). In addition, the recurrence of mild or moderate health effects impose economic challenges on farming households, leading to losses in work productivity, increased expenses for treatment and travel, and potential declines in overall productive capacity (28, 29).

There is little evidence to suggest that the situation regarding pesticide poisoning in the caribbean is disimilar to that observed in other LMICs (18). However, a lack of quality data impedes understanding of the problem and hampers the development of effective strategies for its management. This data deficit leaves regional health professionals and policymakers unaware of the toll of pesticide poisoning on the well-being and productivity of the population. Efforts must be made to generate accurate and comprehensive data on pesticide poisoning to support evidence-based interventions and management of pesticide poisoning in the Caribbean. In an effort to address this data deficit, the aim of this study was to better understand the prevalence of UAPP among smallholder vegetable farmers in Trinidad and Tobago and Jamaica and the pesticides and practices involved.

## 2 Methodology

### 2.1 Study area and farmer survey

**Figure 1:**
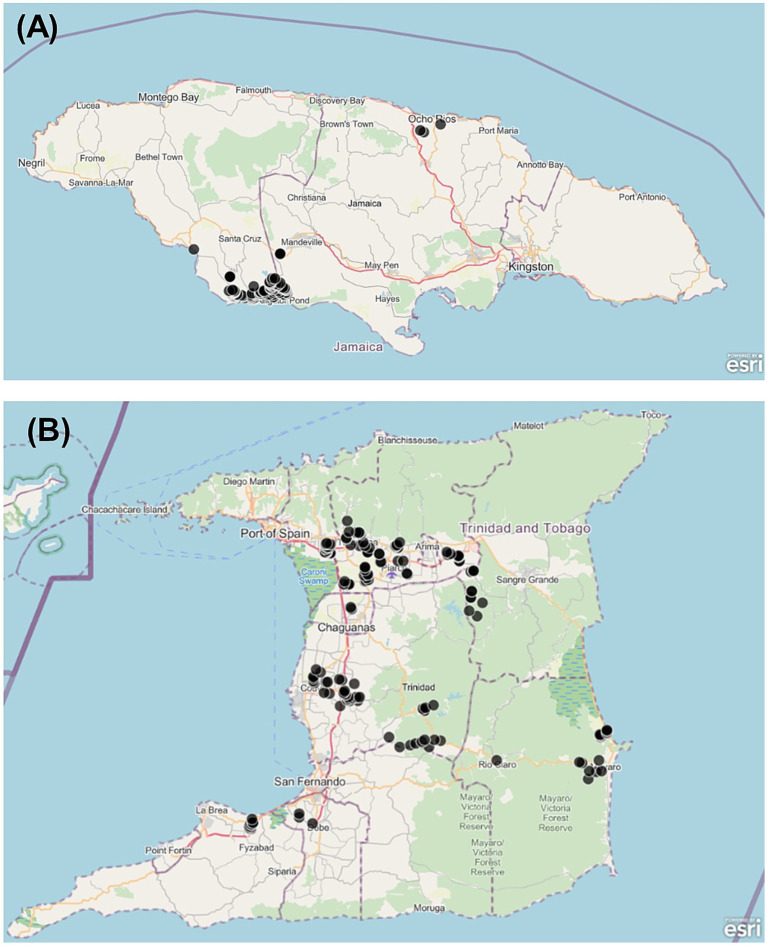
Map of survey locations. **(A)** Jamaica; **(B)** Trinidad. Black dots represent approximate interview locations.

Surveys were conducted in Jamaica and Trinidad and Tobago during January and February 2021. In Jamaica, farming communities were selected in the northern region, in the parish of St Ann, and in the southern region, in the parishes of Manchester and St. Elizabeth (Figure 1). The selection of the communities was based on the high level of vegetable production in these areas. In Trinidad and Tobago, participating farming communites were selected from the Northern, Central and Southern counties of Trinidad island. Lists of registered farmers were provided by all eight counties, and the sample size for each county was proportionate based on the total number of vegetable farmers. In each county, only vegetable growing communities were selected for the survey. In both countries, farmers were selected using convenience sampling, with a target proportion of 20% female. Only farmers that produced vegetables on their own land and applied pesticides were interviewed.

Selected farmers were interviewed individually using a structured questionnaire that was administered via the Tool for Monitoring Acute Pesticide Poisoning (T-MAPP) mobile phone application that was developed by Pesticide Action Network UK (PAN UK). Prior to implementing the survey, the questionnaire was pre-tested with farmers from the selected areas. The results of the pre-test informed minor modifications to some questions to make them more specific to the farming activities and practices in the target communities. Country-specific adaptation also included adding ‘drop lists’ of locally available pesticides, common crops and names of crop pests into the survey questionnaire in order to speed up the surveys and reduce inputting errors. This information was also utilized in the training of members of the survey teams. Respondents were asked for their consent before each interview and the information collected was kept confidential and anonymous.

The information collected during the surveys addressed the crops grown, occurrence of major pest organisms, pesticides use and knowledge, incidence of acute pesticide poisoning and the use of PPE.

The questionnaire had three sections. The first section concerned the farmer characteristics and their conditions of pesticide use. The focus of the second section was incidents of UAPP experienced within the previous 12 months, and the name, formulation and concentration of the pesticide that caused symptoms. Acute pesticide poisoning is variously defined when symptoms occur within 48 (30) or, more conservatively, within 24 h of exposure (31). In this study, UAPP is defined as a symptom or health effect resulting from exposure to a pesticide within 24 h of pesticide exposure.

The final section of the survey concerned the health impacts of the most recent incident of UAPP. All respondents who reported UAPP were asked about symptoms relating to the nervous system and the hematopoietic system. For other organ systems, a triaging system was used to ask questions about affected organ systems only, thereby avoiding asking detailed questions about unaffected organ systems. For each sign and symptom, sub-questions were added to determine its level of severity, derived from a classification tool developed for the International Program on Chemical Safety (30). A total of 330 farmers were interviewed in Jamaica and 199 farmers were interviewed in Trinidad. The survey data was automatically uploaded to a PAN UK managed database which was exported to a csv file for data cleaning and analysis. During data cleaning, two reports were removed from the Trinidad dataset based on incomplete records giving a total of 197 for analysis.

### 2.2 Analysis

Based on the signs and symptoms reported, the severity of UAPP were categorized into mild, moderate and severe, in line with the scoring system proposed by Thundiyil et al. (30). Signs and symptoms which require medical instruments for their assessment (e.g., blood pressure tests), those which cannot be self-reported (e.g., massive haemolysis), or those too open to misinterpretation if assessed by non-medical team staff (e.g., tinnitus or and some cholinergic symptoms) were excluded.

Statistical analyses were performed using SPSS version 25 (SPSS Inc., Chicago, IL, United States). Binary logistic regression analysis was performed to investigate factors influencing the occurrence of UAPP among farmers. Variables entered into the full model were age (14–18, 18–40, 40–60, 60+), gender (male, female), farm size (< 5 ha, > 5 ha), use of PPE (0 items, 1 item, 2–3 items, >3 items), training in PPE use (yes, no), and use of pesticides in original containers with original labels (yes, no). All independent variables were checked for multicollinearity. In relation to the latter variable, some farmers responded that they sometimes use pesticides that were not in original containers. These were treated as a ‘no’ response. However, this variable was excluded for the analysis of the Trinidad data as only one ‘no’ response was recorded. To examine the impact of training on the use of PPE, Fisher’s exact tests were carried out. Use of PPE by an individual was categorized as 0 items, 1 item, 2–3 items, or > 3 items.

## 3 Results

### 3.1 Social demographic characteristics

In total, 527 farmers were interviewed in Jamaica and Trinidad (Table 1). The majority of respondents were men (84%) and almost half were between the ages of 40–60 in both countries.

**Table 1:** Age and gender of the farmers interviewed in Jamaica and Trinidad and Tobago.

		Gender		
Country	Age group	Male *n* (%)	Female *n* (%)	Total *n* (%)
		442 (84)	85 (16)	527 (100)
Jamaica	14–18	2 (1)	0	2 (1)
	18–40	93 (33)	8 (16)	101 (31)
	40–60	129 (46)	32 (64)	161 (49)
	60+	56 (20)	10 (20)	66 (20)
	**Total**	280 (100)	50 (100)	330 (100)
Trinidad	14–18	1 (1)	0	1 (1)
	18–40	57 (35)	12 (34)	69 (35)
	40–60	76 (47)	19 (54)	95 (48)
	60+	28 (17)	4 (11)	32 (16)
	Total	162 (100)	35 (100)	197 (100)

The majority of respondents had farms that were smaller than 5 ha (Jamaica 84%, Trinidad 92%), followed by farm sizes of 5–15 ha (Jamaica 13%, Trinidad 7%) and > 15 ha (Jamaica 3%, Trinidad 2%). All women respondents had a farm size less than 5 ha.

In Jamaica, the most frequently reported crop grown was tomato (57%), followed by watermelon (51%), scallion (36%), cucumber (33%), sweet pepper (26%) and thyme (15%). In Trinidad, the most frequently reported crop grown was hot pepper (42%), followed by sweet pepper (31%), tomato (29%), pumpkin (27%), okra (25%), lettuce (24%), cabbage (22%).

### 3.2 Conditions of use

**Figure 2:**
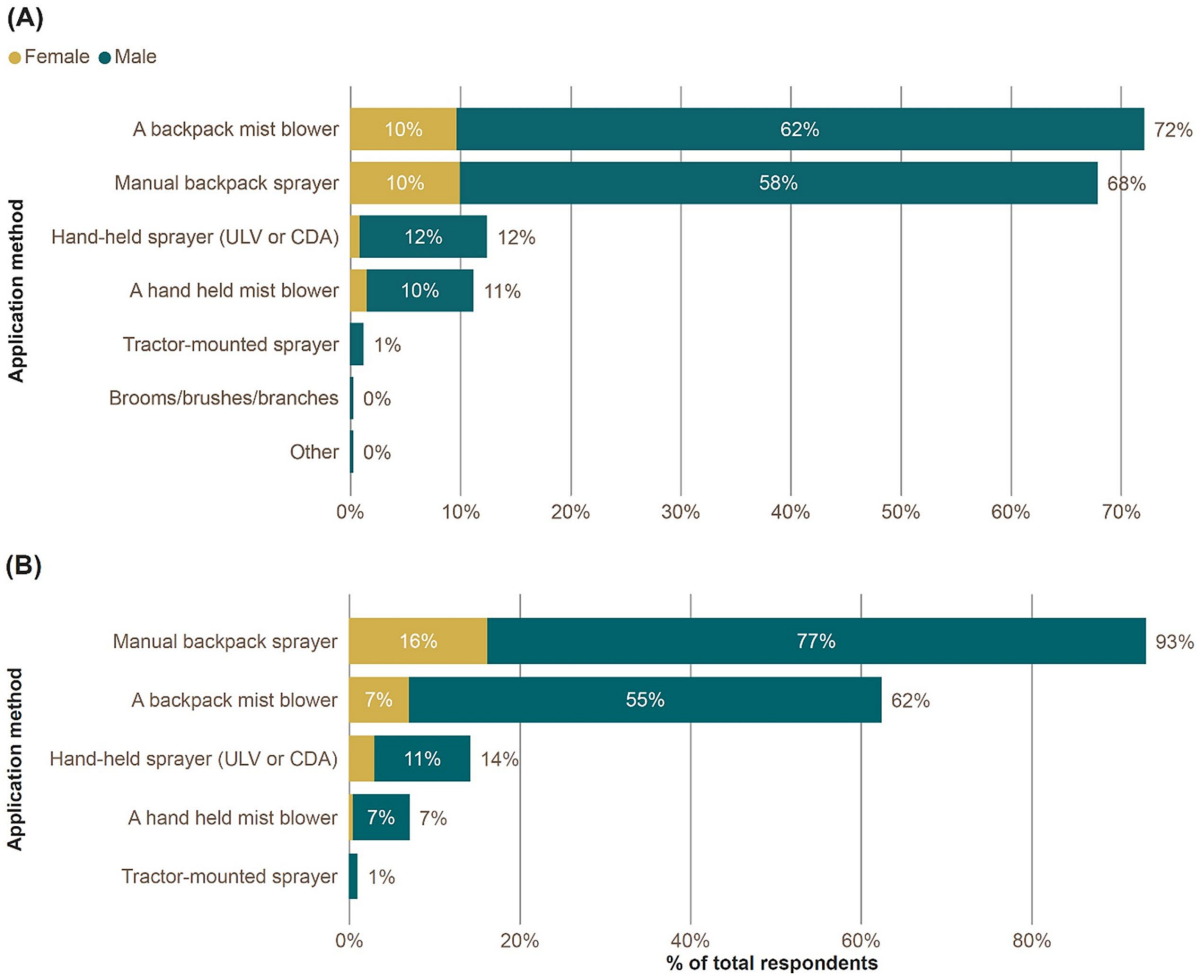
Percentage of total respondents reporting each application method for pesticides. Respondents could report multiple methods of application. Disaggregated by gender. **(A)** Jamaica; **(B)** Trinidad.

The most frequently reported method of pesticide application was a manually operated backpack sprayer with 68% in Jamaica and 93% in Trinidad (Figure 2) followed by a backpack mist blower (Jamaica 72%, Trinidad 62%), hand-held ultra-low volume (ULV) sprayer or controlled droplet application (CDA) sprayer (Jamaica 12%, Trinidad 14%), hand-held mist blower (Jamaica 11%, Trinidad 7%), tractor-mounted sprayer (Jamaica 1%, Trinidad 1%).

**Figure 3:**
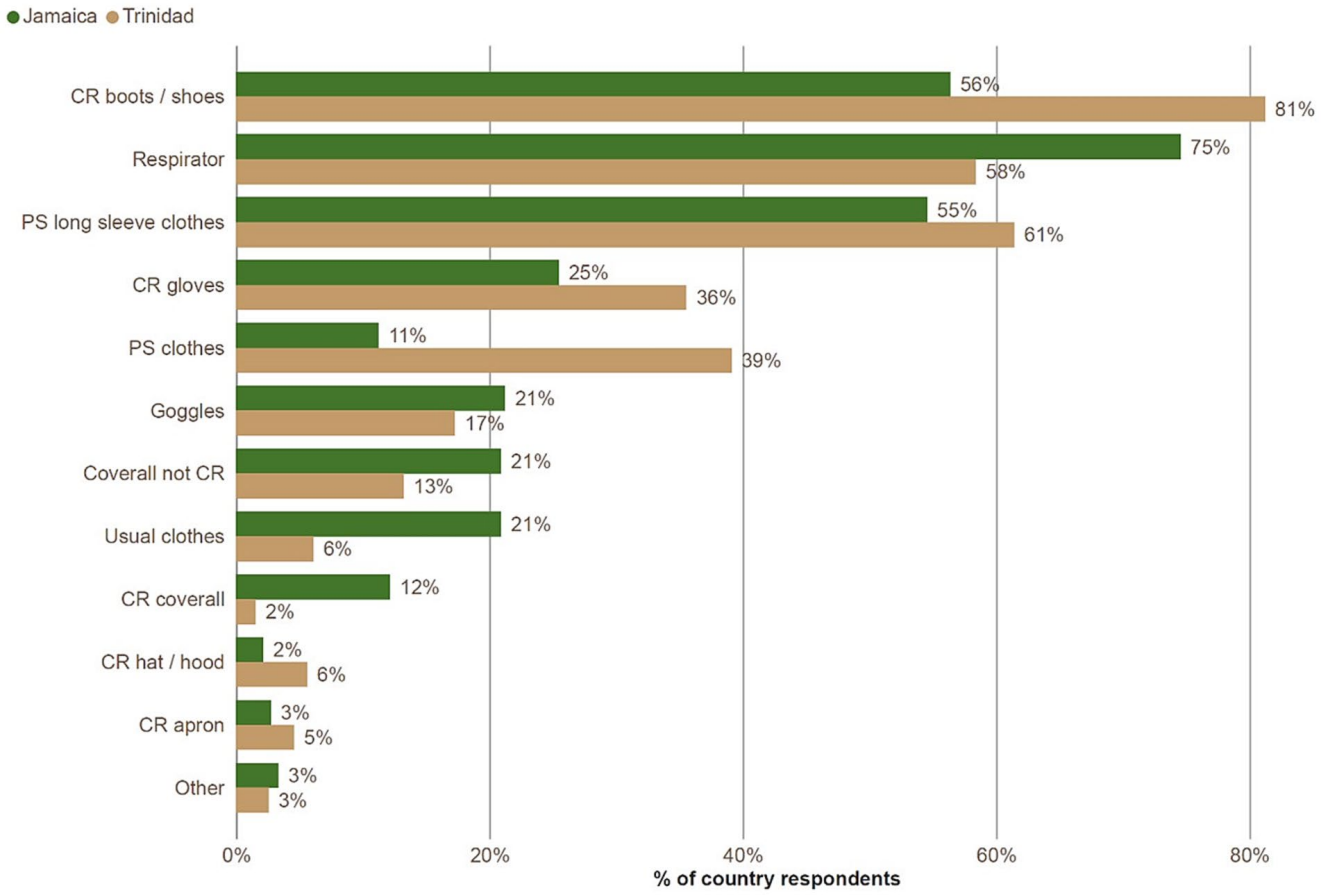
Percentage of respondents reporting items of clothing and PPE worn while handling pesticides, broken down by country. Respondents could report multiple items. Key: CR, chemical resistant; PS, reserved for pesticide spraying.

The most frequently reported items of PPE used while applying pesticides were respirators and chemical resistant boots, followed by chemical resistant gloves (Figure 3). Chemical-resistant coveralls were only used by 12% of respondents in Jamaica and 2% in Trinidad. Non-protective clothing used while spraying pesticides were ‘ordinary clothes with long sleeves or trousers used only for pesticide spraying’, ‘usual clothes’ and ‘ordinary clothes (not long sleeves or trousers) used only for pesticide spraying’.

In Jamaica and Trinidad, 36 and 24% of the respondents, respectively, received training in the use of PPE. In Jamaica, 53% of those trained received training in the previous year, 23% received training 1–2 years ago, and 2% over 10 years ago. In Trinidad, the majority of respondents received PPE training 1–2 years ago (34%), followed by 17% 2–3 years ago. Only 3 respondents (6%) received PPE training in the previous year.

**Figure 4:**
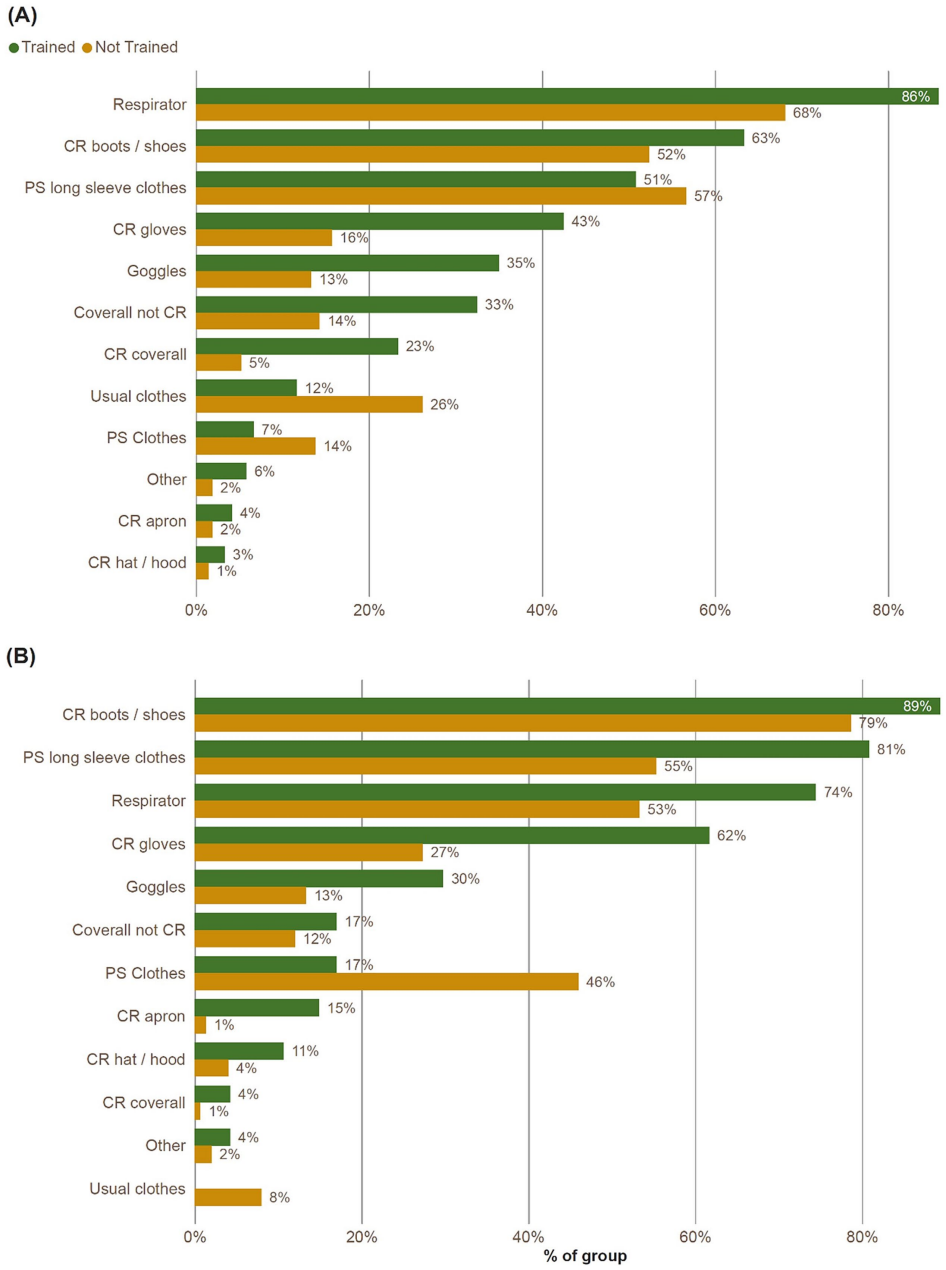
Percentage of clothing and protective equipment worn during handling of pesticides, broken down by respondent training in PPE, reported in **(A)**: Jamaica and **(B)**: Trinidad. Respondents could report multiple items. CR, chemical resistant; PS, reserved for pesticide spraying.

Of the 120 respondents in Jamaica trained in use of PPE, the proportion using protective equipment was higher than those who had not received such training (Figure 4), such as respirator (trained: 86%, not trained: 68%), chemical resistant gloves (trained: 43%, not trained: 16%) and chemical-resistant coverall (trained: 23%, not trained: 5%). The proportion reporting usual clothes was higher for those who were not trained in PPE (26%) compared with those who were trained (12%).

In Jamaica, there was a significant effect of PPE training on PPE use (*p* < 0.001). A higher proportion of trained farmers use more than 3 items of PPE (28%) compared to non-trained farmers (5%). In addition, a lower proportion of trained farmers use no PPE (6%) or 1 item of PPE (20%) compared to non-trained farmers (20 and 30%, respectively; Table 2). In Trinidad there was no significant effect of PPE training on PPE use (*p* = 0.1782).

**Table 2:** Number of PPE items used during pesticide applications by respondents that have received training versus those that have not received training in PPE.

	No. of PPE	Training		No training	
	Items used	n	%	n	%
Jamaica	0	7	6	41	20
	1	24	20	62	30
	2 to 3	56	47	97	46
	>3	33	28	10	5
Trinidad	0	4	9	4	3
	1	15	32	53	35
	2 to 3	18	38	71	47
	>3	10	21	22	15

For the 47 respondents in Trinidad trained in PPE, there was a higher proportion using protective equipment, such as respirator (trained: 74%, not trained: 53%), chemical resistant gloves (trained: 62%, not trained: 27%). The proportion reporting use of long-sleeved ordinary clothes for pesticide application was higher with training (trained: 81%, not trained: 55%), whereas ordinary clothes for pesticide application was higher in the un-trained sample (trained: 17%, not trained: 46%). No PPE trained respondents reported usual clothes, compared to 8% of not trained participants.

### 3.3 Pesticide use

Purchasing pesticides from a farm store was noted by 97% of respondents (100% of women) in Jamaica and 46% in Trinidad. Purchasing from an agricultural supplies store was reported by 61% in Trinidad and 2% in Jamaica. However, it should be noted that in Jamaica, a farm store is characterized as a small local shop and an agricultural supplies store is a much larger shop or retail outlet. Whereas in Trinidad, a farm store is typically characterized as a large shop and an agricultural supplies store is a small shop that only sells agricultural products. Neither sell food products. Less frequently reported include purchasing from neighbors or other farmers (Trinidad 3%), from a contractor (Jamaica 0.3%, Trinidad 2%), extension services (Jamaica 0.6%, Trinidad 0.5%), non-agricultural goods store (Jamaica 0.3%).

In Jamaica, pests and diseases targeted with the most amount of pesticides were whitefly (52% of reports), downy mildew (47%), cutworm (46%), armyworm (39%), beet armyworm (26%), powdery mildew (25%), stink bugs (24%), late blight (19%), thrips (15%), aphids (15%), leaf miners (15%) and early blight (13%).

In Trinidad, pests and diseases targeted with the most amount of pesticides were whitefly, (64% of reports), weeds (56%), leaf miners (42%), thrips (36%), aphids (35%), pinworm (35%), armyworm (34%), broad leaved weeds (26%), grass weeds (26%), cutworm (23%), wilt (21%), rot (20%), mealybugs (18%), anthracnose (16%), leaf spot (16%), scale insects (15%), stink bugs (14%) and damping off disease (13%).

### 3.4 Unintentional acute pesticide poisoning

In Jamaica and Trinidad, 16% (*n* = 330), and 48% (*n* = 197) of the respondents, respectively, reported experiencing acute health impacts within 24 h of pesticide exposure in the last 12 months, but the difference was not significant (Table 3). Following binary logistic regression analysis, none of the following variables; i.e. age, gender, farm size, use of PPE, training in PPE use and the use of pesticides in original containers with original labels, had a significant effect on whether farmers experienced UAPP (*p* < 0.05; Table 3).

**Table 3:** Frequency of unintentional acute pesticide poisoning experienced by farmer respondents in Jamaica and Trinidad over the previous 12 months disaggregated by variables entered into binary logistic regression models.

Variable	Category	Men		Women		Total		
		n	% UAPP	n	% UAPP	n	% UAPP	*p*-value
Jamaica								
Gender	Men					280	15	0.415
	Women					50	22	
Age	14–40	95	14	8	13	103	14	0.293
	40–60	129	19	32	25	161	20	
	>60	56	11	10	20	66	12	
PPE training	Yes	99	15	21	10	120	14	0.459
	No	181	15	29	31	210	18	
PPE	0 Items	42	19	6	17	48	19	0.972
	1 Item	70	14	16	19	86	15	
	2–3 Items	129	16	24	25	153	17	
	>3 Items	39	13	4	25	43	14	
Farm size	<5 ha	230	16	48	23	278	17	0.393
	>5 ha	50	12	2	0	52	12	
Pesticide in original containers	Yes	245	16	48	23	293	17	0.644
	No	35	14	2	0	37	14	
Trinidad								
Gender	Men					162	48	0.931
	Women					35	49	
Age	14–40	58	48	12	50	70	49	0.759
	40–60	76	51	19	42	95	49	
	>60	28	36	4	75	32	41	
PPE training	Yes	41	49	6	50	47	49	0.876
	No	121	47	29	48	150	47	
PPE	0 Items	7	57	1	100	8	63	0.164
	1 Item	57	40	11	27	68	38	
	2–3 Items	74	51	15	73	89	55	
	>3 Items	24	50	8	25	32	44	
Farm size	<5 ha	146	47	35	49	181	49	0.826
	>5 ha	16	50	0	0	16	50	

**Figure 5:**
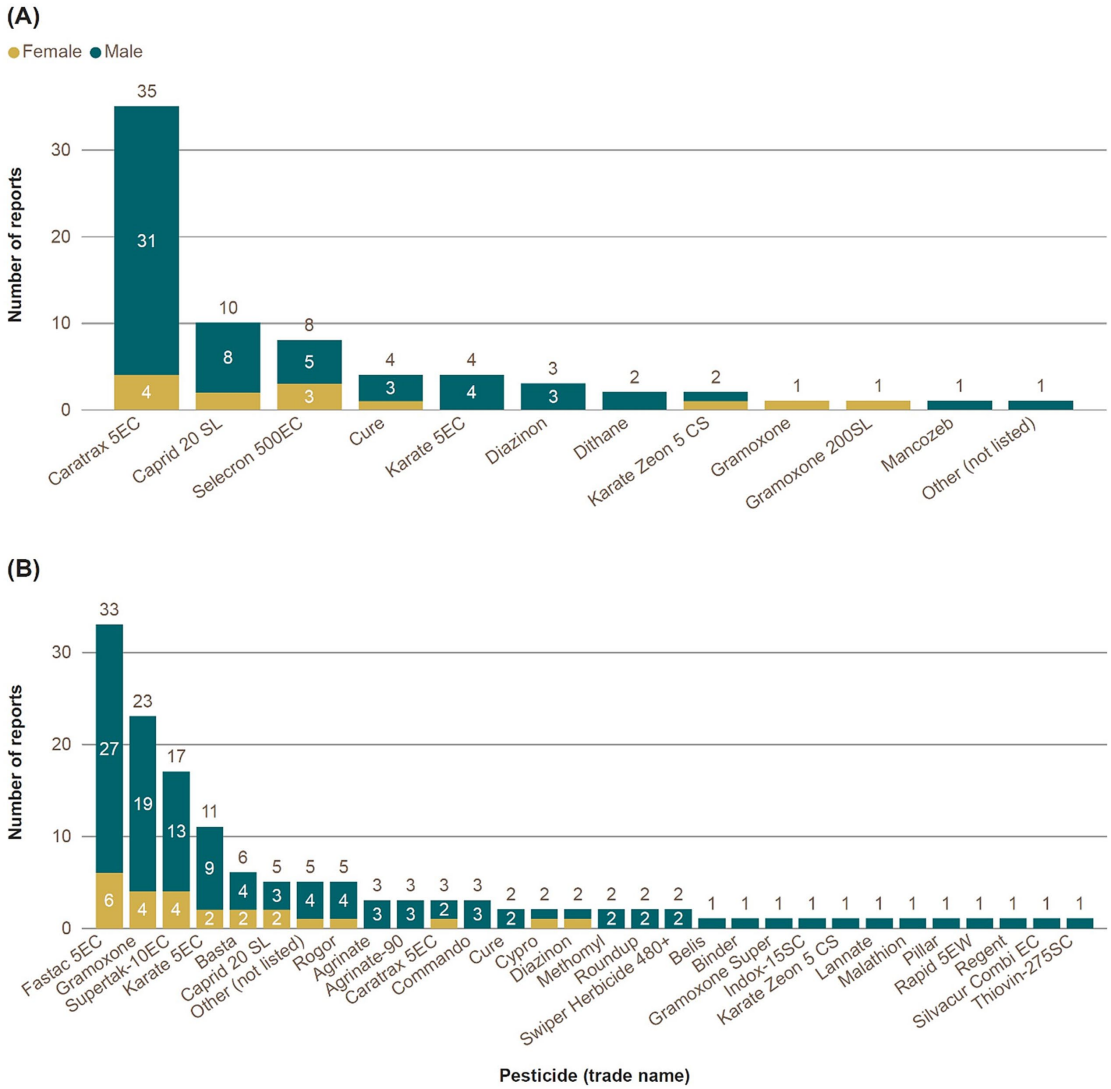
Frequency of pesticide trade names reported by respondents in **(A)** Jamaica and **(B)** Trinidad experiencing acute pesticide poisoning symptoms in the last 12 months. Disaggregated by gender.

Of those experiencing UAPP in the previous 12 months, the most frequently reported pesticide associated with an UAPP incident in Jamaica was lambda-cyhalothrin (Caratrax 5E), reported by 65% of respondents (by 72% of men; 36% of women), followed by acetamiprid (Caprid 20 SL; 18.5%; 19% men; 18% women) and profenofos (Selecron 500EC; 14.8%; 12% men; 27% women; Figure 5). Lambda-cyhalothrin also was the most frequently reported pesticide involved in the most recent incident of poisoning (61.5%), followed by profenofos (11.5%), and acetamiprid (9.6%; Supplementary Figure 1).

In Trinidad, the most frequently reported pesticide involved in an UAPP incident over the previous 12 months was alpha-cypermethrin, reported by 53% of respondents reporting poisoning incidents. The alpha-cypermethrin products associated with these incidents were Fastac 5EC, reported by 35% of men and 35% of women reporting poisoning incidents, and Supertak-10EC, reported by 17% men and 24% women (Figure 5). Paraquat (Gramoxone: 25%; 25% men; 24% women) was the second most frequently reported pesticide involved in an UAPP over the previous 12 months. Alpha-cypermethrin (i.e., Fastac 5EC: 35% and Supertak-10EC: 11%) was also the most frequently reported pesticide involved in the most recent incident of poisoning, followed by Gramoxone (paraquat: 18%; Supplementary Figure 1).

**Figure 6:**
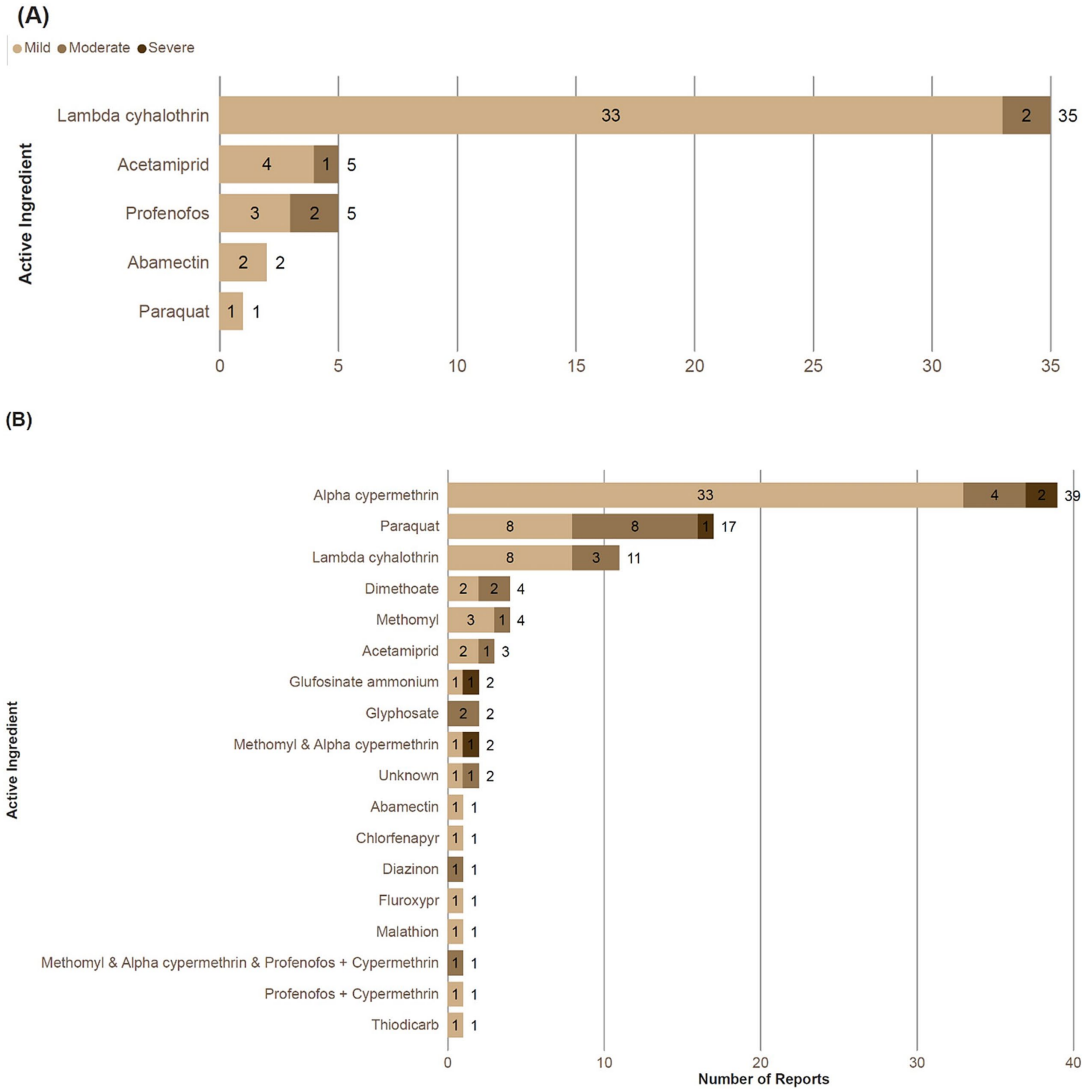
Severity score assigned to most recent acute pesticide poisoning incidents in **(A)** Jamaica and **(B)** Trinidad by active ingredient. Key: ‘+’ represents co-formulation of different active ingredients in one product; ‘&’ represents different products with different active ingredients reported as a mixture.

Of the 54 persons reporting poisoning incidents in Jamaica, two declined to answer further questions on symptoms and four individuals did not report symptoms of UAPP. Thus, a severity score was calculated for 48 respondents (Figure 6). Of these, 90% were classified as mild incidents, and 10% as moderate. Of the moderate incidents, two (40%) were associated with lambda-cyhalothrin, two (40%) with profenofos and one (20%) with acetamiprid.

Of the 94 poisoning incidents in Trinidad, the majority were classified as mild (69%), followed by moderate (26%) and severe (5%). Of the five severe incidents, two were associated with exposure to alpha-cypermethrin and one with paraquat, one with glufosinate ammonium (Basta) and one with a mixture of the pesticides methomyl (Agrinate-90) and alpha-cypermethrin (Supertak 10EC). A pesticide mixture involving three products (i.e methomyl, alpha-cypermethrin and profenofos + cypermethrin co-formulation) also was associated with a moderate poisoning incident.

All respondents who reported UAPP were then asked about their symptoms relating to the nervous system and the hematopoietic system, with other organ systems selected by respondents affected, the results of which are presented in Table 4. In Jamaica, respondents frequently reported local effects on the skin (56%) followed by symptoms impacting respiratory system (25%) and local effects on the eyes (10%; Supplementary Figure 2). In Trinidad, the main symptoms reported were local effects on the eyes (50%), local effects on the skin (44%), respiratory system (22%) and gastrointestinal system (15%; Supplementary Figure 3).

**Table 4:** Frequency and percentage of responses to body systems reported to have been affected within 24 h of using pesticides.

		Gender		
		Male *n* (%)	Female *n* (%)	Total *n* (%)
Jamaica	Nervous System	43 (100.0)	9 (100.0)	52 (100.0)
	Hematopoietic System	43 (100.0)	9 (100.0)	52 (100.0)
	Local effects on the skin	28 (65)	1 (11)	29 (56)
	Respiratory System	11 (26)	2 (22)	13 (25)
	Local effects on the eyes	3 (7)	2 (22)	5 (10)
	Metabolic balance	1 (2)	0	1 (2)
	Muscular System	0	1 (11)	1 (2)
	Gastro-intestinal System	0	0	0
	Cardiovascular System	0	0	0
	Renal System	0	0	0
Trinidad	Nervous System	77 (100.0)	17 (100.0)	94 (100.0)
	Hematopoietic System	77 (100.0)	17 (100.0)	94 (100.0)
	Local effects on the skin	33 (43)	8 (47)	41 (44)
	Respiratory System	16 (21)	5 (29)	21 (22)
	Local effects on the eyes	36 (47)	11 (65)	47 (50)
	Metabolic balance	6 (8)	1 (6)	7 (8)
	Muscular System	4 (5)	0	4 (4)
	Gastro-intestinal System	11 (14)	3 (18)	14 (15)
	Cardiovascular System	0	0	0
	Renal System	0	0	0

**Figure 7:**
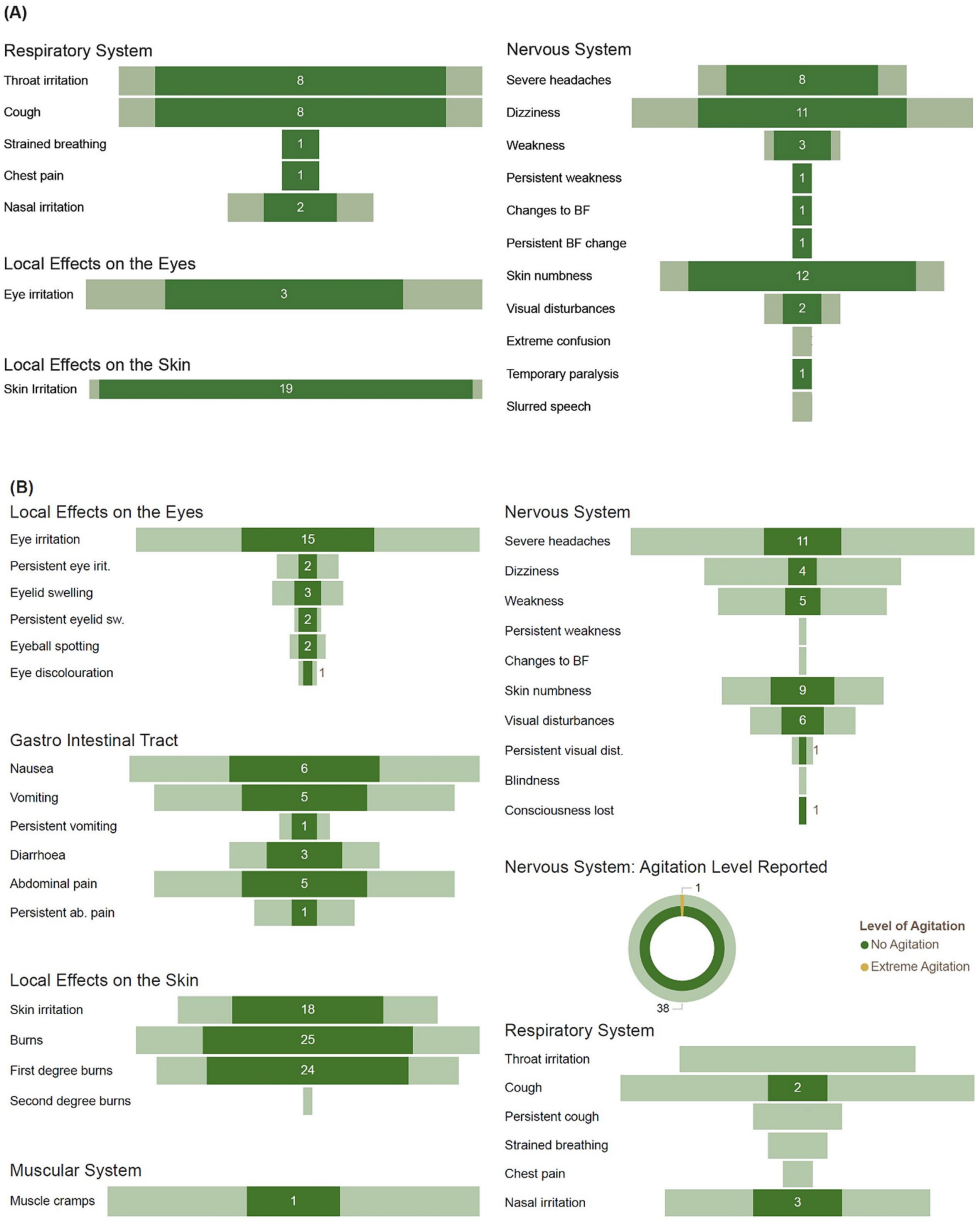
The frequency of symptoms reported following **(A)** lambda cyhalothrin exposure (dark shaded) as a proportion of symptoms reported for all UAPP incidents (light shaded) by respondents in Jamaica. **(B)** alpha cypermethrin exposure (dark shaded) as a proportion of symptoms reported for all UAPP incidents (light shaded) by respondents in Trinidad. Key: Eye irit. Relates to eye irritation; eyelid sw. relates to eyelid swelling; ab. Pain relates to abdominal pain; chest pain related to a sharp stabbing pain in your chest which feels worse when you cough; Weakness includes any of the following symptoms: slowness or weakness when carrying out routine tasks, difficulty in walking or with balance, tremors or shaking, abnormal involuntary posture, abnormal movements of the tongue, jaw, face, arms, legs, neck or trunk; Changes to BF includes any of the following symptoms: increased or decreased salivation, decreased sweating, difficulty in urinating, constipation; Persistent relates to symptoms present for 48 h or more.

The most frequently reported symptoms during UAPP incidents associated with lambda-cyhalothrin in Jamaica (*n* = 38) were skin irritation (50%), nervous system effects, abnormal skin tingling or numbness (32%), dizziness (29%), severe headache (21%), muscle weakness and abnormal involuntary movements (8%), visual disturbances (5%), and temporary paralysis (3%). Respiratory system effects, throat irritation (21%), cough (21%) and nasal irritation (5%) (Figure 7).

Symptoms reported for lambda cyhalothrin related UAPP incidents in Trinidad (*n* = 11) were nervous system effects, severe headache (55%), dizziness (36%), muscle weakness and abnormal involuntary movements (36%), visual disturbances (27%), abnormal skin tingling or numbness (9%; Supplementary Figure 4). Local effects on the skin, irritation (46%), burns (46%). Effects on the eyes, irritation (36%), respiratory system effects, cough (18%), throat irritation (9%).

In Trinidad, symptoms reported following alpha cypermethrin exposure (*n* = 38, Figure 7) included local effects on the skin, burns (64%) and irritation (46%) Effects on the eyes, irritation (39%), persistent irritation for more than 48 h (5%), eyelid swelling (8%), persistent swelling for more than 48 h (5%). Nervous system effects, severe headaches (28%), abnormal skin tingling or numbness (23%), visual disturbances (15%), dizziness (10%), muscle weakness and abnormal involuntary movements (10%), loss of consciousness (3%) and extreme agitation (3%).

Symptoms frequently reported following paraquat exposure in Trinidad (n = 17) were nervous system effects, severe headache (65%), dizziness (29%), muscle weakness and abnormal involuntary movements (29%), abnormal skin tingling or numbness (29%), visual disturbances (18%; Supplementary Figure 5). Effects on the eyes, irritation (35%), persistent eye irritation (12%), eyelid swelling (18%). Respiratory system effects, cough (29%), persistent painful cough for more than 48 h (18%), throat irritation (24%), nasal irritation (24%). Skin burns (24%), irritation (12%). Gastro intestinal effects nausea (12%), vomiting (12%), abdominal pain (12%). Muscle cramps and stiffness not from physical labor (12%) and fever (12%), persistent fever (6%).

For incidents associated with acetamiprid, frequently reported symptoms in Trinidad (*n* = 3) were nervous system effects, severe headache (100%), dizziness (100%), muscle weakness and abnormal involuntary movements (67%) and eye irritation (100%). In Jamaica (*n* = 5), these were nervous system effects, such as dizziness (60%), severe headache (40%) and extreme confusion or hallucinations (20%). Followed by eye irritation (20%), skin irritation (20%), gastrointestinal effects, nausea (20%) and abdominal pain (20%; Supplementary Figure 6).

In Jamaica, frequently reported symptoms following profenofos exposure (*n* = 6), included nervous system effects, dizziness (50%), abnormal skin tingling or numbness (50%), severe headache (17%; Supplementary Figure 7). Respiratory system effects included cough (33%), nasal irritation (33%), throat irritation (17%).

**Figure 8:**
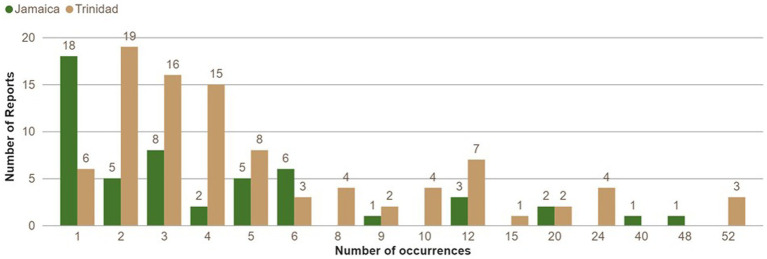
Number of acute pesticide poisoning incidents reported in the last 12 months, disaggregated by country.

In Jamaica, the number of occurrences of acute pesticide poisoning incidents per individual over the previous 12 months ranged from one to 48. In Trinidad, the reported occurrences per individual ranged from one to 52 (Figure 8). Most respondents reported experiencing 1–5 occurrences (Jamaica 71%, Trinidad 68%), followed by 6–10 occurrences (Jamaica 13%, Trinidad 14%) and more than 10 (Jamaica 16% Trinidad 18%).

Medical assistance was sought by 7% (four) of respondents reporting UAPP in Jamaica. Two visited a health practitioner and one a hospital, with all three reporting that pesticides were mentioned in the diagnosis. One further respondent reported visiting a clinic.

In Trinidad 22% reporting UAPP sought medical treatment. The most frequently visited destination for treatment was a health practitioner (48% of respondents, *n* = 21), followed by a hospital (38%), a family member (10%) and a traditional healer (5%). Eighteen of the 21 diagnoses mentioned pesticides.

In Jamaica, respondents reported missing two to 14 days off work in the previous 12 months due to UAPP, totaling 35 work days missed over 12 months. This equates to 65 work days lost per year for every 100 farmers experiencing UAPP. In Trinidad, the number of work days missed due to UAPP ranged from one to 36, totaling 122 work days missed over 12 months. This equates to 130 work days lost per year per 100 farmers experiencing UAPP.

## 4 Discussion

The findings from this study revealed a high incidence of occupational pesticide exposure amongst vegetable farmers in Jamaica and Trinidad. Of the farmers surveyed, 16 and 48% experienced symptoms of UAPP within the previous 12 months, respectively. These rates highlight a consistent trend with previous studies of UAPP in Jamaica (17) and for similar crops in other countries (32–36) and are in line with global estimates of UAPP among farmers and farm workers (19). Furthermore, the majority of UAPP incidents reported in this study were associated with relatively few active ingredients, i.e., lambda-cyhalothrin, alpha-cypermethrin, paraquat, acetamiprid and profenofos. Aside from acetamiprid, all of these are considered to be highly hazardous pesticides (HHPs) according to the PAN HHP list (37), with specific concerns for lambda-cyhalothrin and paraquat due to their documented hazards to human health (37, 38).

### 4.1 Pesticides of concern

The two most frequently reported pesticides associated with incidents of UAPP were synthetic pyrethroid insecticides, lambda-cyhalothrin (Caratrax 5EC, Karate 5EC, Karate Zeon 5CS; mostly in Jamaica) and alpha-cypermethrin (Fastac 5EC, Supertak 10EC; in Trinidad), representing 59% of all the cases of UAPP.

Lambda cyhalothrin is a synthetic pyrethroid insecticide that is highly toxic to mammals, fish, aquatic invertebrates and honeybees and has a CLP Regulation (Classification, Labeling and Packaging Regulation (EC) No 1272/2008) classification of H330 (fatal if inhaled) (38). Several studies have also identified lambda-cyhalothrin as potential endocrine disrupter (39, 40). Acute poisoning symptoms following exposure to lambda-cyhalothrin include dermal, neurological, respiratory, ocular, and gastrointestinal symptoms, fever and muscle aches (41). Paresthesia (tingling/ prickling in the skin) has also been reported frequently, particularly on the face. Other symptoms reported include lack of appetite and fatigue (42). Seizures, convulsions, coma, non-cardiogenic pulmonary oedema may occur in severe cases (43). Furthermore, there is no known antidote. The reported effects are comparable with our findings from 38 incidents reported in Jamaica and 11 incidents in Trinidad relating to lambda-cyhalothrin products. A similar range of effects (primarily dermal, neurological, respiratory, ocular and gastrointestinal) were reported with dermal effects being the most common.

Other incidents of UAPP from lambda-cyhalothrin have been reported worldwide (44). A survey of coffee and vegetable farmers in Arumeru District, Arusha region, Tanzania found that lambda-cyhalothrin was among the pesticides most associated with poisoning even though only around a fifth of respondents reported using it (45). In the US, a relatively large number of lambda-cyhalothrin incidents are reported in EPA’s Incident Data System (IDS) and SENSOR-Pesticides. In 2023, the IDS reported 21 incidents caused by lambda-cyhalothrin of which most were classified as ‘moderate’ and two as ‘major’ (46). As of February 28, 2017, Canada had identified 95 human incidents involving lambda-cyhalothrin, mostly during use or on re-entry into sprayed areas (47).

In 2017, two formulations of lambda-cyhalothrin containing lambda-cyhalothrin 50 g/L (EC) and 50 g/L (CS) were notified to the Rotterdam Convention by Georgia as proposals for Severely Hazardous Pesticide Formulations (document UNEP/FAO/RC/CRC.13/16). These notifications were based on surveys of farmers and farm workers conducted in 2016. Of 497 people surveyed, lambda-cyhalothrin was associated with 25% of the 61 incidents of UAPP reported where the respondent named the product; higher than any other product reported. Eight of these incidents related to Karate 5 EC. In addition, in 2021, various uses of lambda-cyhalothrin and associated products were prohibited in Canada including in lettuce and bulb vegetables (48). Within the EU, lambda-cyhalothrin is listed as a candidate for substitution because the Acceptable Operator Exposure Level (AOEL) is significantly lower than those of the majority of the approved active substances within the group of insecticides (Commission Implementing Regulation (EU) 2015/408). In the US, lambda-cyhalothrin is a restricted use insecticide (49) and in 2020, the US EPA identified risks to operators and proposed prohibiting several types of application including backpack foliar sprays on vegetables and trees and mechanically pressurized handgun foliar sprays on typical field crop uses (50).

Alpha-cypermethrin is also a synthetic pyrethroid insecticide that is highly toxic to mammals, fish, aquatic invertebrates and honeybees (38). It is banned in at least 29 countries worldwide (51). Acute alpha-cypermethrin poisoning can produce adverse health effects similar to lambda-cyhalothrin and other pyrethroids, such as dermal irritations, mild neurological symptoms and gastrointestinal symptoms. Increasingly severe cases can result in more serious neurological effects such blurred vision, an increase in sweating, uncontrollable muscle fiber twitching and palpitations (41, 52). These symptoms are comparable with symptoms reported by 39 individuals in relation to alpha-cypermethrin poisoning in this study, with the most frequently reported effects being dermal irritation and skin burns followed by other symptoms such as muscle weakness and abnormal involuntary movements, severe headaches, nausea, visual disturbances and extreme agitation.

Worldwide there are few reports in the literature of poisoning with alpha-cypermethrin. However, there are more reported cases of poisoning for the enantiomerically-impure cypermethrin formulations (53–55), which has qualitative similarities in toxicity and metabolism to alpha-cypermethrin. As of 2024, there are 2 formulations of cypermethrin (10% EC and 35% EC) notified as severely hazardous pesticide formulations and scheduled for review by the Chemical Review Committee of the Rotterdam Convention.

The US EPA IPS database, includes nine incidents related to alpha-cypermethrin, with the majority classified as moderate impacts on human health and one fatality reported in 2018 (46). The IPS database contains a more comprehensive list of incidents related to cypermethrin with 40 reports in 2023, the majority classified as moderate impacts on human health, and one major report.

In this study, paraquat, a non-selective herbicide, was frequently associated with UAPP in Trinidad. Paraquat has been banned in over 67 countries due to its human and environmental harms (56). Due to its high toxicity and lack of an effective treatment, it is most frequently linked to fatal poisoning incidents following ingestion of very small quantities (57). Furthermore, paraquat is also known to cause poisoning via exposure through skin or inhalation (58). Acute poisoning with paraquat can have adverse effect on the eyes, and cause nosebleeds. Dermal exposure can lead to skin irritation and burns, and absorption through the skin can lead to severe lung and kidney damage (59, 60). These symptoms align with the reports from 17 individuals in Trinidad of eye irritation (35%), cough (29%), persistent and painful cough (18%), skin numbness (29%) and burns (24%). A study of farmworkers in Colombia found that those who were chronically exposed to paraquat had an increased prevalence of self-reported asthma (36).

Two further pesticides that were frequently associated with UAPP in this study were the insecticides, acetamiprid and profenofos. Acetamiprid, reported in both countries, is a neonicotinoid insecticide which has moderate mammalian toxicity, and has been reported to cause ill health effects in humans. Previously reported symptoms include negative effects to the gastrointestinal system, central nervous system, cardiovascular and respiratory system (61). Respondents in this survey mainly reported severe headaches and dizziness with extreme confusion and visual disturbances.

Profenofos, banned in at least 34 countries (51), was associated with UAPP in Jamaica. It is an organophosphate insecticide that is extremely toxic to humans, as a result, it is listed as a candidate chemical that is scheduled for review by the Chemical Review Committee of the Rotterdam Convention. It inhibits the enzyme acetylcholinesterase, resulting in harmful effects on the central nervous system, peripheral nervous system and respiratory function (62, 63). A recent review of epidemiological studies on pesticide exposure in Latin America and the Caribbean also found consistent evidence of an association between exposure to organophosphate pesticides, including profenofos, and acute and chronic health effects, including respiratory and allergic effects (18). This aligns with what was reported in this survey, with respondents reporting profenofos to cause throat and nasal irritation, coughing and central nervous system effects, dizziness, skin tingling or numbness and slurred speech.

In Trinidad there were several reports of poisoning following exposure to mixtures of pesticides, some of which were co-formulations in the same product eg. Cypro (profenofos + cypermethrin), others were multiple products reported to cause poisoning together eg. Agrinate-90 and Supertak-10EC (methomyl & alpha-cypermethrin). The toxicity of mixtures can be affected in three ways: independent, dose additive and interactive, the latter of which can be synergistic or antagonistic (64). Synergistic effects have been found for pesticide coformulations containing organophosphate and pyrethroid insecticides, such as the profenofos + cypermethrin co-formulation reported in Trinidad (41). The acetylcholinesterase inhibition exhibited by the organophosphate (in this case, profenofos) results in a reduced rate of metabolism of pyrethroids (in this case, cypermethrin) to their non-toxic metabolites (41, 65), potentially resulting in more severe cases with more intensive treatment required (66–68). In this study, cases related to a mixture of methomyl, a carbamate, and alpha-cypermethrin, a pyrethroid (Agrinate-90 and Supertak-10 EC) were reported, one incident was classified as severe. While there are no synergistic effects identified in the active ingredients’ modes of action, both pyrethroids and carbamates have synergistic effects with the adjuvant piperonyl butoxide (64). This is a non-pesticidal additive that increases the toxicity of insecticides by inhibiting metabolism of the active ingredient (64). However, it can be difficult to identify products containing piperonyl butoxide due to its common inclusion on the label as a non-specific ‘inert ingredient’ for confidential and protected information purposes (69). Due to the complex toxicological effects caused by exposure to mixtures of pesticides, co-formulants and adjuvants, further studies are needed to understand the various impacts on human health (18).

### 4.2 Economic consequences of UAPP

In both countries surveyed in this study, only a small proportion of farmers reporting UAPP sought medical treatment for their symptoms. In Trinidad, 21 individuals (22% of those reporting UAPP) sought medical treatment, 47% of which were treated by a health practitioner and 38% went to a hospital. Whereas in Jamaica, 4 individuals (7% of those reporting UAPP) sought medical assistance from either a health practitioner, clinic or hospital.

These findings indicate that only a small proportion of farmers experiencing UAPP report ill health effects to health services, thus highlighting the importance for surveillance systems to monitor the impacts of pesticides on rural communities. Another study in Tanzania reported a similarly low proportion of UAPP being reported to a health care facility (19%) (70). The authors of that study identified a number of reasons for this that may also apply to farmers in the Caribbean. These include (i) inability to afford medical bills; (ii) the mild severity of the symptoms; (iii) anticipated difficulties in diagnosis and treatment; (iv) distance/ poor access to health care facility, and (v) unawareness of the long-term adverse health effects of pesticides. This suggests the need for monitoring systems that complement the data collected by hospitals and healthcare facilities to fully understand the level of UAPP and pesticides involved (70).

Of the respondents reporting UAPP in Jamaica and Trinidad, 13 and 17%, respectively, reported taking days off work as a result of their symptoms. The period off work ranged from 1 to 36 days. These days of missed work represents a significant negative impact of UAPP on human health as well as causing potential production and economic losses to the farmers (71), with impacts on wider society. For example, the economic cost of UAPP in the United States of America is estimated to be over $1 billion per year (72) and in 37 sub-Saharan countries, this was estimated to cost US$4.4 billion in 2005 (27). In the Philippines, a study modeling the impact of pesticide use on productivity indicated that a reduction in insecticide use would result in a net improvement in productivity, which the authors attributed to improved farmer health (73).

### 4.3 Conditions of use

The results from the statistical analysis revealed that age, gender, PPE use, training in PPE, presence of original label or farm size had no significant effect on whether farmers experienced UAPP. Of particular importance, these findings indicate that the use of PPE did not significantly reduce the likelihood of exposure to pesticides. Although PPE is frequently promoted as central to pesticide exposure risk reduction measures, there are questions around its efficacy in offering adequate protection for pesticide applicators. Current regulatory mechanisms for approval of many highly hazardous pesticides rely on protection by PPE, and adequate labels describing appropriate protective equipment to prevent the adverse health effects of handling and applying pesticides (74). This reliance on protective equipment results in many products being approved for use in countries where effective use of PPE is hindered by factors outside the direct control of the farmers and farm workers. For instance, high costs, inadequate supply and lack of information regarding the recommended item (74, 75). Regardless of adequate access to PPE, there are other barriers to use, notably in warm climates such as the Caribbean using full PPE is uncomfortable and potentially an additional danger to health due to potential dehydration and heat stroke (74, 76). Women face further challenges due to poorly fitting PPE designed for men, cultural expectations of dress and economic barriers to purchasing (25).

PPE training programs are often considered a central element to reducing the harms caused by occupational exposure to pesticides. In our study we found low rates of PPE training in both countries, 35% reported training in Jamaica and 24% in Trinidad. Farmer training programs may cover a range of issues, including the health hazards of pesticides and suitable personal protection. However, farmers that access such training may still lack information about safer alternatives. The FAO/ WHO recommend that ‘Guidance on the selection and use of PPE should be included in farmer and public health training programs by extension or advisory services (77). Training should also include use of non-chemical methods of pest management as the first option or selection of less hazardous products (such as biological pesticides) that require less PPE, in particular if access to or wearing PPE is not realistic. In a review of the role of PPE in pesticide risk prevention, Garrigou et al. (74) report that training interventions are difficult to link to an increase in PPE use, although there are some studies with increased use of particular items of PPE. There remains to be seen consistent uptake of full, recommended PPE as a result of training interventions. This is reflected in our survey results, where training had significant effects on PPE use in Jamaica, with no statistical significance in Trinidad. For example, there was a reduction in the proportion of individuals that had received training reporting wearing ‘usual clothes’ when applying pesticides. However, there was no significance in the use of PPE on reducing the likelihood of UAPP in both countries. Further, there are also considerations around correct reporting and use of PPE items. A study of smallholder coffee farmers in Jamaica highlighted discrepancies between reported use and observed behavior with PPE items (4).

A further challenge to the efficacy of protective clothing and equipment comes from maintenance of contaminated items. In both countries surveyed, respondents frequently reported the use of usual clothes or ordinary clothes reserved for pesticide spraying. However, previous studies have identified pesticide residues in such clothing, even after cleaning, that can be then be absorbed by skin (78). Furthermore, the level of contamination in clothing and PPE can also be affected by the inert ingredients present within formulations, either through increasing pesticide permeability or reducing the effectiveness of cleaning (69).

### 4.4 Pests targeted by HHPs and available alternatives

Tomato and sweet pepper were commonly cultivated by the surveyed farmers in both countries. The other most common crops grown were watermelon in Jamaica and hot pepper in Trinidad. The pests most frequently targeted with pesticides were whitefly, lepidoptera larvae, downy mildew and weeds. Whitefly aphids (*Bemisia tabaci*) are a common sucking pest of tomato, pepper, watermelon and leafy vegetables in the Caribbean and in addition are vectors of plant viruses such as tomato yellow leaf curl virus (TYLCV) and cucumber mosaic virus (8, 79). Lepidopteran larvae, such as armyworm (*Spodoptera* spp.), are also common pests of tomato, pepper, watermelon and leafy vegetables in the region. The main uses of lambda-cyhalothrin, alpha-cypermethrin, acetamiprid and profenofos (four out of five of the most frequently reported pesticides associated with incidents of UAPP in this study) include control of lepidopteran larvae and whitefly. Paraquat, one of the other pesticides frequently linked to UAPP in this study, is typically used against weeds in vegetable farming (56).

As well as causing harms to human health, many HHPs affect vital ecosystem services essential for sustainable production, such as pollination, nutrient cycling, and natural pest control (80). Furthermore, many HHPs harm predators and parasitoids of insect pests and can lead to a ‘resurgence’ of pest populations (due to their faster reproduction rates and shorter life cycles), an escalating cycle of pesticide use, and further loss of natural pest control (the so-called pesticide treadmill) (6, 7). Fortunately, when following the ecologically-based principles of agroecology or Integrated Pest Management (IPM), there is an array of tools and techniques that are safer and more sustainable than HHPs (56, 81–83). These include cultural techniques, biological control, physical methods, varietal resistance, use of ‘non-synthetic’ chemicals, and use of less hazardous synthetic chemicals as a last resort. We provide examples of these in Table 5.

**Table 5:** Selected studies providing relevant examples of alternatives to HHPs for lepidopteran larvae and whitefly pest management in vegetables grown under tropical conditions.

Method	Example	Country	References
Cultural control	Tomatoes intercropped with onion or garlic had reduced levels of whiteflies and aphids.	Uganda	Tumwine (102)
	Cabbage intercropped with black mint or thyme had reduced damage and higher yields in comparison with cabbage monocrops.	Jamaica	Robinson (103)
	An African marigold intercrop reduced both *Helicoverpa armigera* eggs and caterpillars in the adjacent tomato with a consequent reduction in the number of bored fruits from 57 to 6%.	India	Ibrahim et al. (104)
	Chilli intercropped with French bean or amaranth produced higher chilli yields than chilli cropped alone.	India	Anitha and Geethakumari (105); Innazent et al. (106)
Biological control	About 700,000 ha of pastures, cassava and vegetables were estimated to be protected from lepidoptera pests in 2003 via the release of a *Trichogramma* species of wasp.	Cuba	Van Lenteren and Bueno (107)
	Regular release of *Trichogramma species* effectively controlled lepidopteran pests in tomato, chilli and cabbage.	India	Krishnamoorthy (108)
Biopesticdes	A commercial neem biopesticide was as effective as lambda-cyhalothrin against insect pests of callaloo and pak choy, with no significant difference in harvestable and marketable yields.	Jamaica	McDonald et al. (112)
	Chilli pepper plots treated with commercial biopesticides containing neem or *Bt* significantly reduced infestation levels of major insect pests, such as whitefly (*B. tabaci*), and improved yields compared to untreated control plots.	Ghana	Adom et al. (109)
	Cucumber plots treated with commercial biopesticides containing neem, *B. bassiana*, *Lecanicillium lecanii*, or *Metarhizium anisopliae* significantly reduced whitefly (*B. tabaci*) eggs, nymphs and adults, and improved yields compared to untreated control plots. Also, the mean yield of the neem treatment was no different to the chemical treatment.	India	Ghongade and Sangha (110)
Integrated package	Integrated control strategies evaluated against *H. armigera* were found more effective than any single measure of control.	India	Krishnamoorthy (108)
	A compilation of simple, affordable, and environmentally sound IPM strategies in tropical vegetable crops.	Global	Srinivasan (82)

There are several examples of successful cultural techniques used for vegetable pest management in the tropics to create or maintain unfavorable conditions for pests (82, 84, 85). For example, crop rotation with non-host plants can break pest population cycles and the carryover of diseases from one season to the next and intercropping with plants that have allelopathic properties can repel insect pests (86, 87).

One of the most important elements of agroecology and IPM includes the conservation of natural enemies of pests, based on the principle of enhancing beneficial biological interactions (83). One crucial step toward achieving this aim is to avoid both calendar-based spraying and broad-spectrum insecticides. In addition, biological control of pests may be enhanced by attracting predatory insects into the crop or by augmentative mass releases of artificially reared parasitoids or predators. One successful method to attract predatory insects into the crop foliage is the food spray method, with proven success in smallholder cotton in Benin and Ethiopia (88, 89) and more recently in tomato and onion farms in Ethiopia (90). Other methods include incorporating flowering plants or other crops, such as alfafa, within fields or field margins to provide sources of food and refuge for natural enemies (90–92).

Biopesticides are also proven effective alternatives to HHPs (93, 94). These include microbial pesticides (bacteria, viruses, fungi), botanical pesticides (e. g. neem, *Azadarachta indica*) and biochemical pesticides (e.g., pheromones and plant volatiles) (81). Examples of microbial pesticides used for vegetable pests are preparations of nuclear polyhedrosis virus (NPV), the bacterium *Bacillus thuringiensis* (Bt), and the fungus Beauveria bassiana. Commercial products of both *B. bassiana* (e.g., Biopower^®^, Botanigard^®^, Mycotrol^®^ and Naturalis^®^) and Bt (e.g., Agree^®^, Bactivec^®^, Dipel^®^) are currently registered across the Caribbean, including Jamaica and Trinidad and Tobago. In Trinidad, the pest control potential of *Bacillus amyloliquefaciens* also was demonstrated against vegetable pathogens in lettuce as a model system and proposed as an alternative to synthetic fungicides (95). Botanical pesticides are also a well tried and tested in many traditional agricultural systems. For example, extract of the seed or leaves of neem is a well-known and widely used botanical in vegetable IPM that is available in the Caribbean and is effective against several insect pests in vegetables (81, 96). Also, recent research in Trinidad investigating the efficacy of seaweed extracts for tomato and sweet pepper disease management found significantly fewer incidences of bacterial spot and early blight in foliar sprayed plants (97).

Overall, the key strategy to successfully reduce reliance on pesticides and eliminate the use of HHPs for insect, weed and disease management is to use an integrated approach that uses a combination of physical, cultural, and ecological techniques that are selected based on the location-specific context (56, 92). Practical training, demonstrations and participatory field trials with experiential learning are also important to help farming communities engage and fully understand the hazards of HHPs and build their knowledge and confidence in using alternative methods (90, 98, 99).

### 4.5 Limitations

This survey was limited to farmers only. Thus, we did not capture the effects of pesticide exposure to employed farm workers (who do not own farms) or other members of the farming household who are also exposed to pesticides (18). For example, as well as pesticide exposure during application, exposure can occur via deliberate or accidental consumption, pesticide drift, the preparation of pesticide mixtures or the cleaning or wearing of pesticide-contaminated clothing (25).

We acknowledge that retrospective studies are subject to recall bias. However, evidence suggests that using a 12 month recall period can lead to a fair degree of reliability (100). For example, a study by Gabbe et al. (101) on self-reported sports injuries showed that survey participants are able to recall the number of injuries and the body region affected with a high degree of accuracy over a 12 month recall period when a clear and context-specific definition of the injury/symptom is provided (as was provided in our questionnaire). Overall, however, the study concluded that recall bias over 12 months is likely to lead to a conservative estimate of the scale of injury.

Unfortunately, our questionnaire did not disaggregate farm sizes below five ha. This limited the level of analysis we are able to conduct to determine the effect of farm size on UAPP. Future studies in smallholder agricultural systems should thus include a more granular assessment of farm size.

## 5 Conclusion and recommendations

This study highlights the significant issue of occupational pesticide exposure among vegetable farmers in Jamaica and Trinidad. The percentage of Jamaican (16%) and Trinidadian (48%) farmers reporting symptoms associated with UAPP in the previous 12 months emphasizes the ongoing risks associated with pesticide use in agriculture and underscores the persistent nature of this problem. A substantial proportion of UAPP incidents were linked to a few highly hazardous pesticides, particularly lambda-cyhalothrin, acetamiprid, and profenofos in Jamaica, and alpha-cypermethrin, paraquat and lambda-cyhalothrin in Trinidad. Given the well-documented adverse effects of these chemicals, particularly lambda-cyhalothrin and paraquat, on human health, the incidents of UAPP in this study should be of significant concern to health authorities in Jamaica and Trinidad. This clearly indicates an urgent need for improved regulation and safer alternatives to these pesticides. The data gathered from this study provided essential evidence to the authorities to help them make informed decisions to withdraw the registration of alpha-cypermethrin and paraquat dichloride active ingredients in Trinidad and Tobago with an intent of protecting the health of farmers. Thus, highlighting the value of such post-registration surveillance information.

Despite the widespread promotion of PPE as a key mitigation strategy, this study found no significant correlation between PPE use and a reduced incidence of UAPP in Jamaica and Trinidad. This reveals the limitations of PPE and training in mitigating pesticide exposure. This finding calls into question the efficacy of current regulatory mechanisms that rely heavily on PPE for protection and suggests the need for more effective and practical interventions. The economic consequences of UAPP are also potentially substantial, with significant workdays being lost due to pesticide-related illnesses, impacting both individual farmers and the broader agricultural economy.

Based on the findings of this study, there is a clear need to withdraw the registrations for hazardous pesticides that are causing serious harm to farmer health so that they are no longer available, enhance pesticide poisoning surveillance systems, including biomonitoring, strengthen regulatory frameworks, and provide information and training to farmers on the implementation of alternative pest management strategies, including the promotion of integrated approaches to pest management and sustainable agricultural practices. This approach must aim to reduce reliance on HHPs, thereby improving both human health and environmental sustainability, and promoting long-term agricultural productivity in Jamaica, Trinidad and Tobago, and the wider Caribbean.

## Data Availability

The raw data supporting the conclusions of this article will be made available by the authors, without undue reservation.
