# Supplementary material for: Assessment of unintentional acute pesticide poisoning among smallholder vegetable farmers in Trinidad and Jamaica
Source: Front Public Health. 2024 Nov 5;12:1470276. doi: 10.3389/fpubh.2024.1470276 (PMC11573769; doi:10.3389/fpubh.2024.1470276)
Supplement: Supplementary file 1 [file Data_Sheet_1.docx]

Supplementary Material

# Supplementary Figures and Tables

## Supplementary Figures


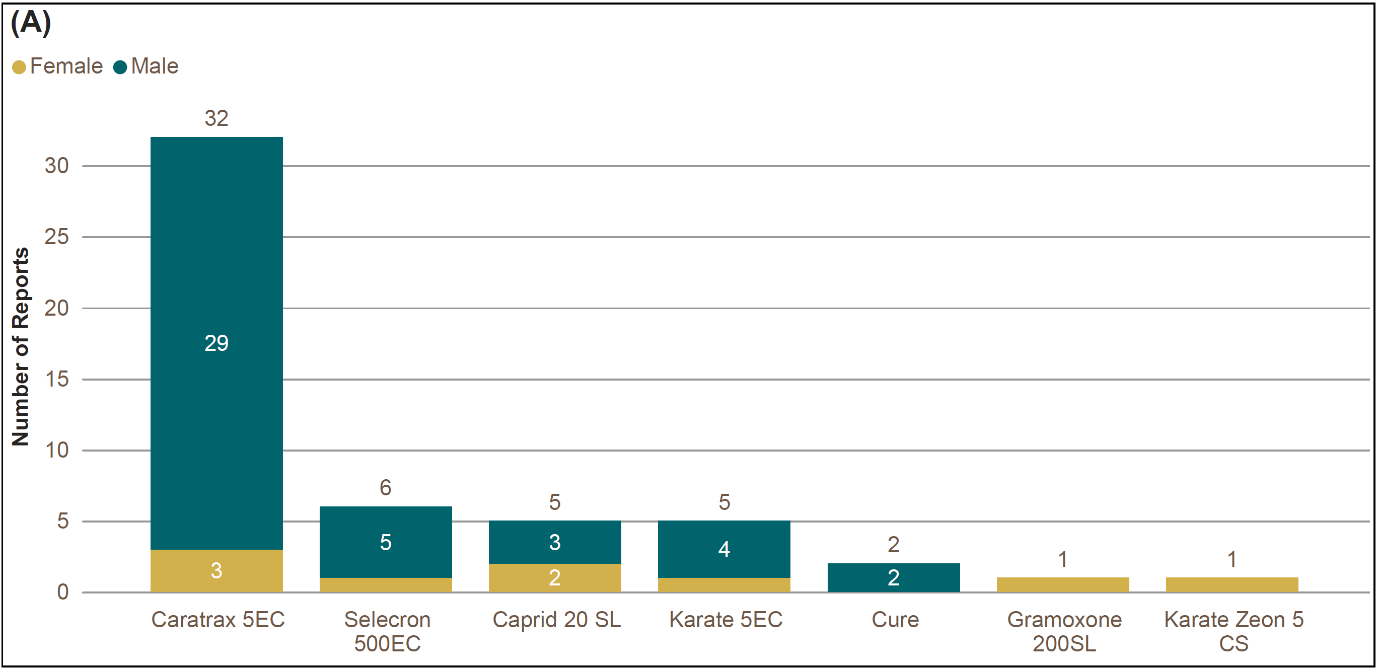

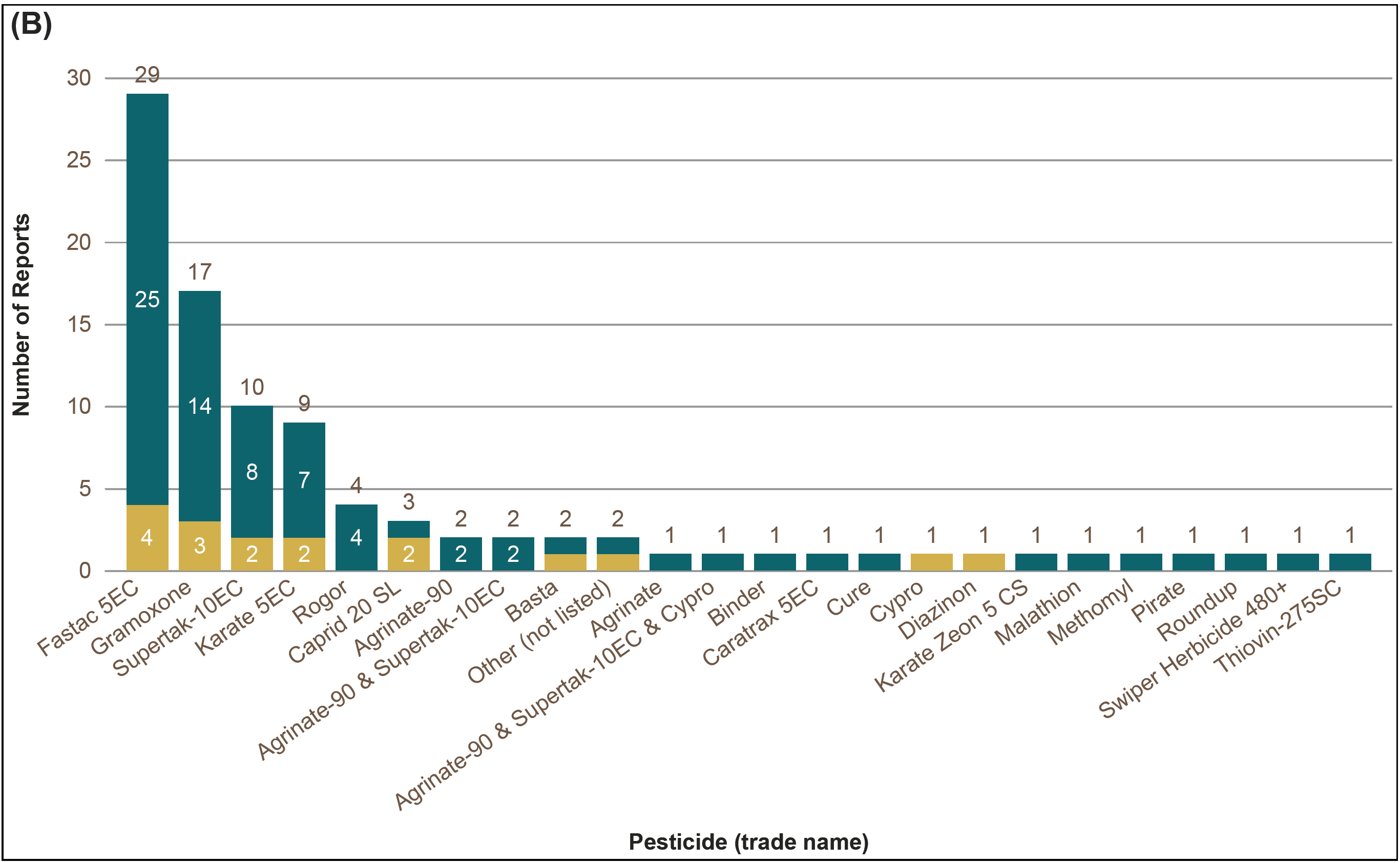


Supplementary Figure 1: Frequency of pesticide trade names reported by respondents in **(A)** Jamaica and **(B)** Trinidad, that caused the most recent incident of acute pesticide poisoning symptoms in the last 12 months. Disaggregated by gender.


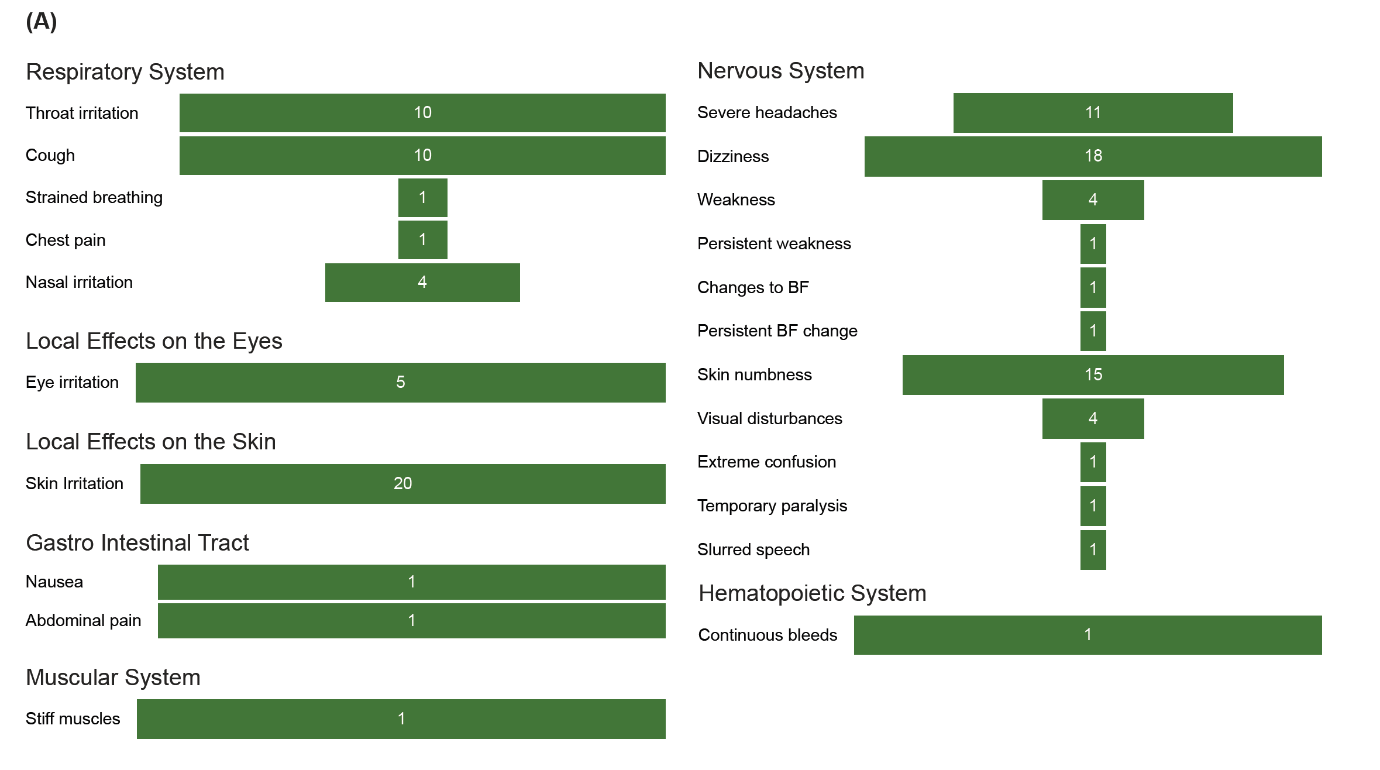
Supplementary Figure 2: The frequency of all symptoms reported following reports of UAPP in Jamaica (n=52). Key: **Eye irit.** relates to eye irritation; **eyelid sw.** relates to eyelid swelling; **ab. Pain** relates to abdominal pain; **chest pain** related to a sharp stabbing pain in your chest which feels worse when you cough; **Weakness** includes any of the following symptoms: slowness or weakness when carrying out routine tasks, difficulty in walking or with balance, tremors or shaking, abnormal involuntary posture, abnormal movements of the tongue, jaw, face, arms, legs, neck or trunk; **Changes to BF** includes any of the following symptoms: increased or decreased salivation, decreased sweating, difficulty in urinating, constipation; **Persistent** relates to symptoms present for 48 hours or more.


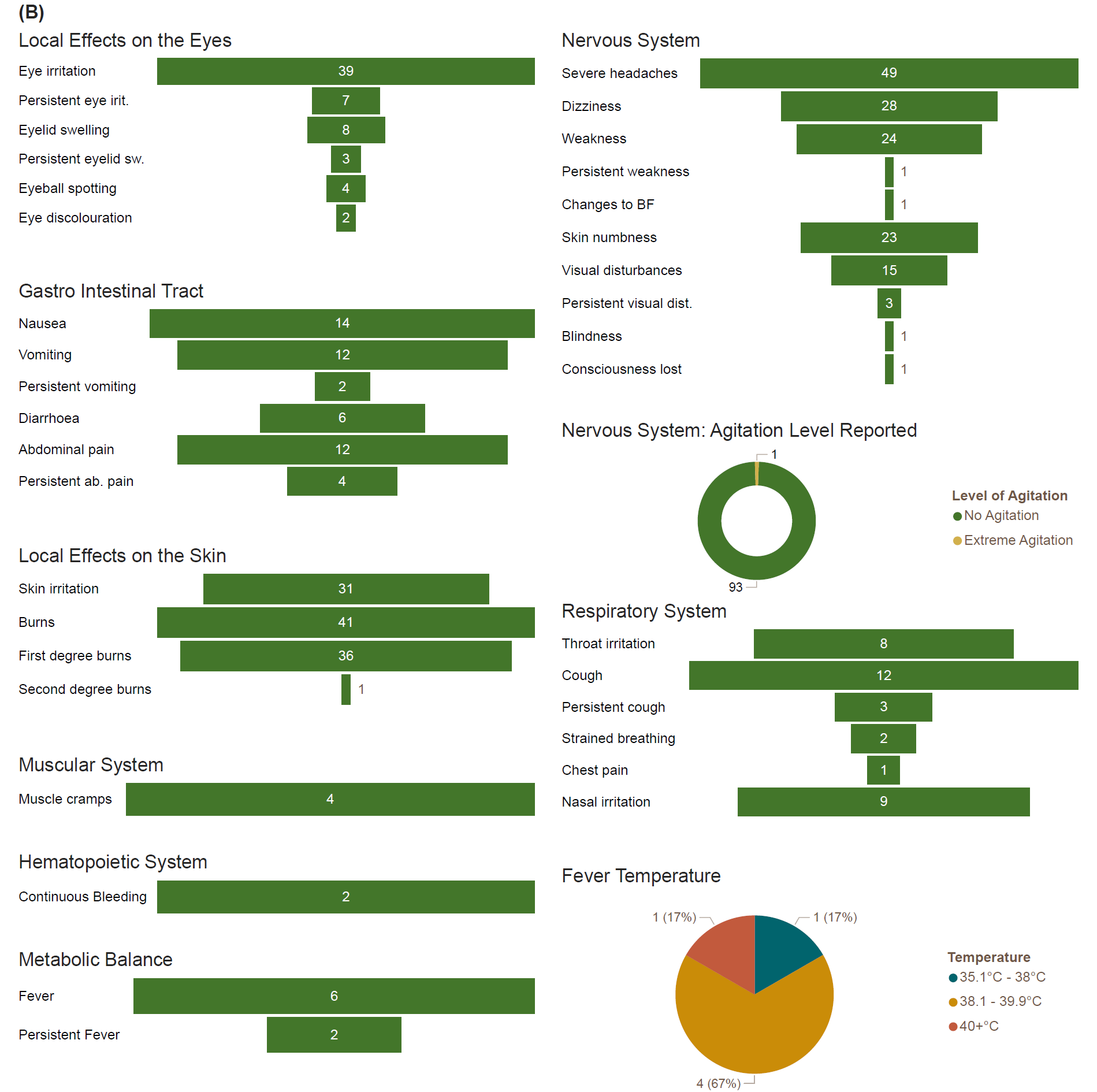
Supplementary Figure 3: The frequency of all symptoms reported following reports of UAPP in Trinidad (n=94). Key: **Eye irit.** relates to eye irritation; **eyelid sw.** relates to eyelid swelling; **ab. Pain** relates to abdominal pain; **chest pain** related to a sharp stabbing pain in your chest which feels worse when you cough; **Weakness** includes any of the following symptoms: slowness or weakness when carrying out routine tasks, difficulty in walking or with balance, tremors or shaking, abnormal involuntary posture, abnormal movements of the tongue, jaw, face, arms, legs, neck or trunk; **Changes to BF** includes any of the following symptoms: increased or decreased salivation, decreased sweating, difficulty in urinating, constipation; **Persistent** relates to symptoms present for 48 hours or more.

Supplementary Figure 4: The frequency of symptoms reported following reports of UAPP by lambda-cyhalothrin in Trinidad (n=11). Key: **Eye irit.** relates to eye irritation; **eyelid sw.** relates to eyelid swelling; **ab. Pain** relates to abdominal pain; **chest pain** related to a sharp stabbing pain in your chest which feels worse when you cough; **Weakness** includes any of the following symptoms: slowness or weakness when carrying out routine tasks, difficulty in walking or with balance, tremors or shaking, abnormal involuntary posture, abnormal movements of the tongue, jaw, face, arms, legs, neck or trunk; **Changes to BF** includes any of the following symptoms: increased or decreased salivation, decreased sweating, difficulty in urinating, constipation; **Persistent** relates to symptoms present for 48 hours or more.


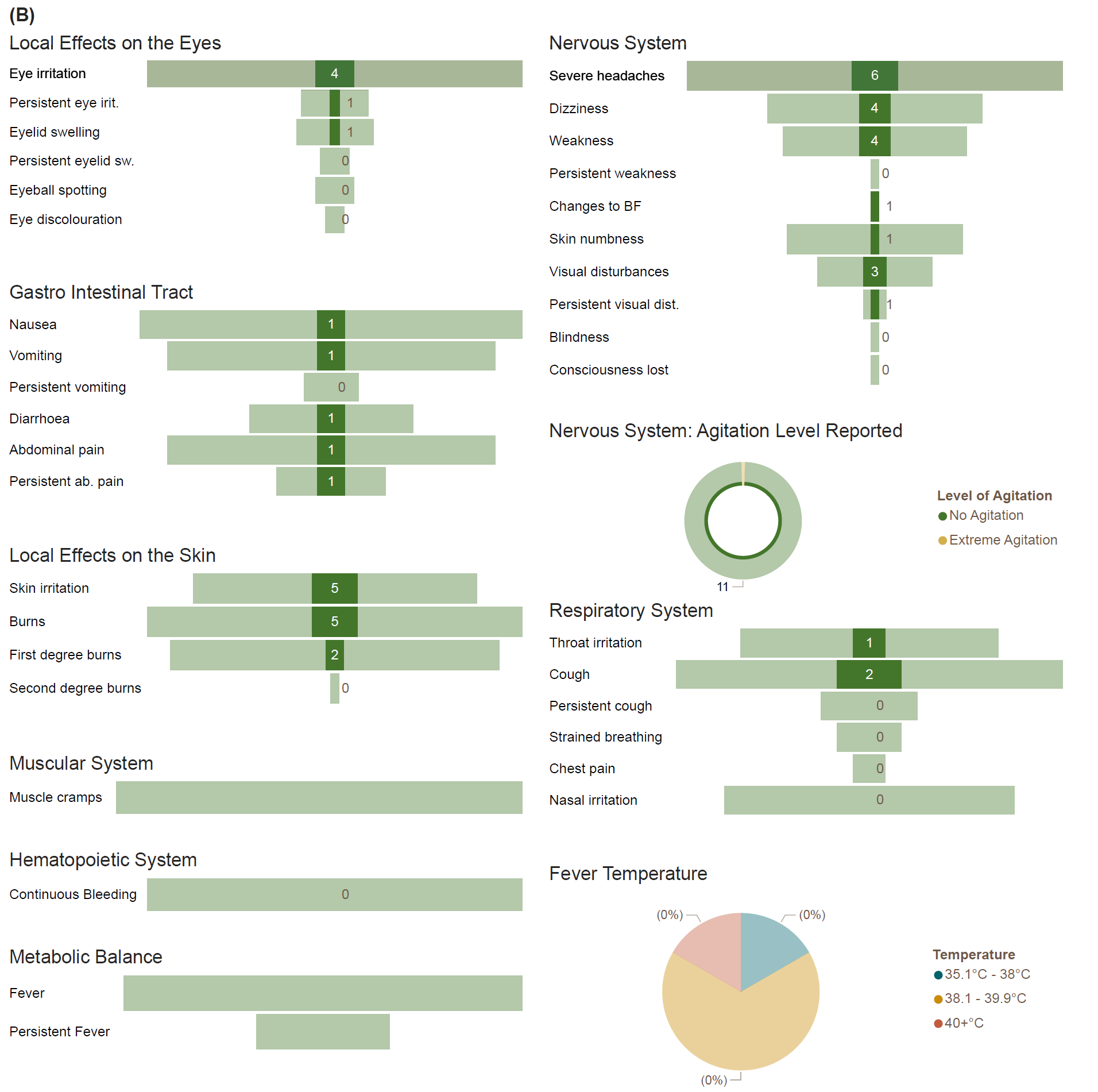


Supplementary Figure 5: The frequency of symptoms reported following reports of UAPP by paraquat in Trinidad (n=17). Key: **Eye irit.** relates to eye irritation; **eyelid sw.** relates to eyelid swelling; **ab. Pain** relates to abdominal pain; **chest pain** related to a sharp stabbing pain in your chest which feels worse when you cough; **Weakness** includes any of the following symptoms: slowness or weakness when carrying out routine tasks, difficulty in walking or with balance, tremors or shaking, abnormal involuntary posture, abnormal movements of the tongue, jaw, face, arms, legs, neck or trunk; **Changes to BF** includes any of the following symptoms: increased or decreased salivation, decreased sweating, difficulty in urinating, constipation; **Persistent** relates to symptoms present for 48 hours or more.


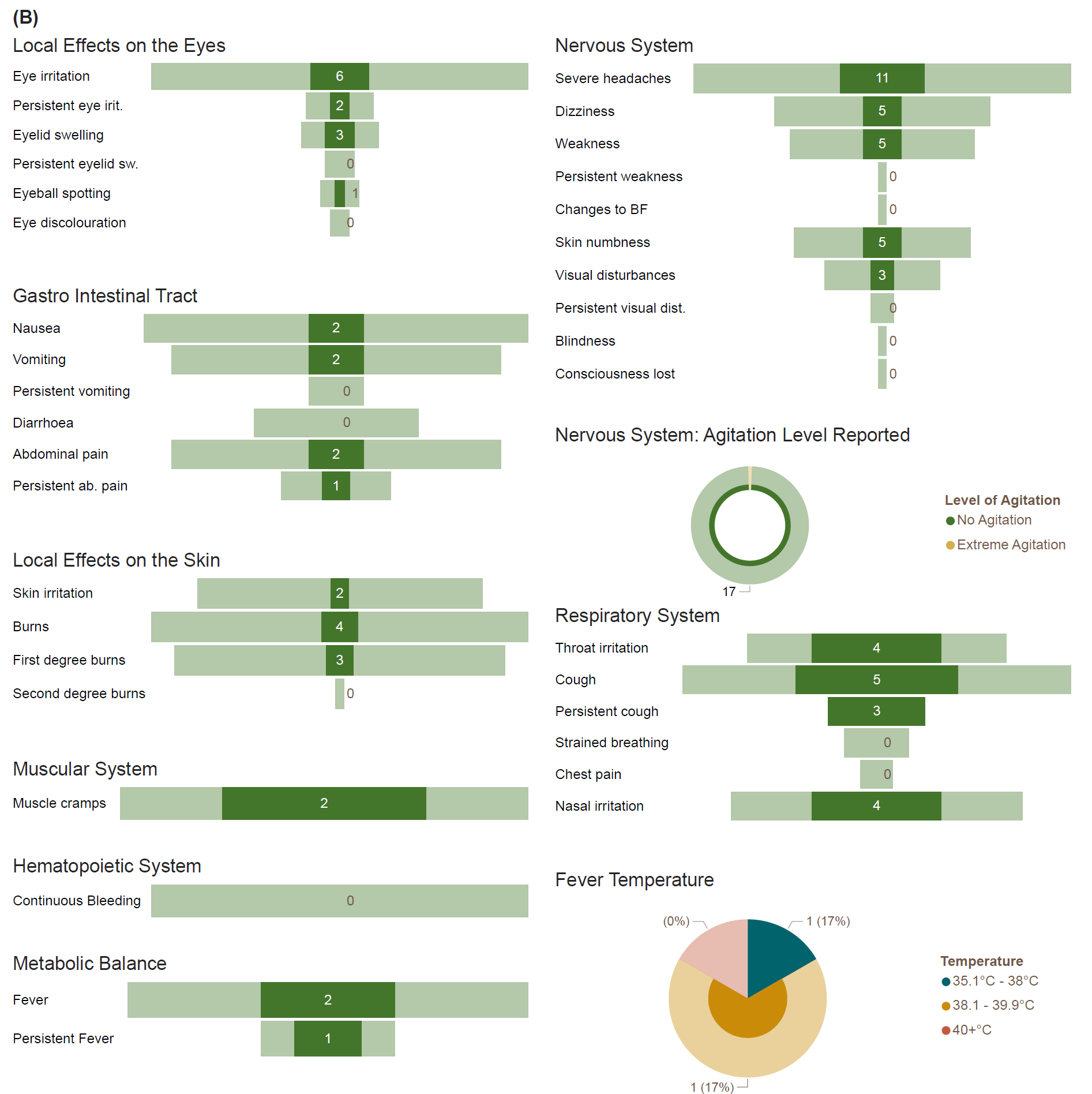


Supplementary Figure 6: The frequency of symptoms reported following reports of UAPP by acetimiprid in Jamaica (n=5). Key: **Eye irit.** relates to eye irritation; **eyelid sw.** relates to eyelid swelling; **ab. Pain** relates to abdominal pain; **chest pain** related to a sharp stabbing pain in your chest which feels worse when you cough; **Weakness** includes any of the following symptoms: slowness or weakness when carrying out routine tasks, difficulty in walking or with balance, tremors or shaking, abnormal involuntary posture, abnormal movements of the tongue, jaw, face, arms, legs, neck or trunk; **Changes to BF** includes any of the following symptoms: increased or decreased salivation, decreased sweating, difficulty in urinating, constipation; **Persistent** relates to symptoms present for 48 hours or more.


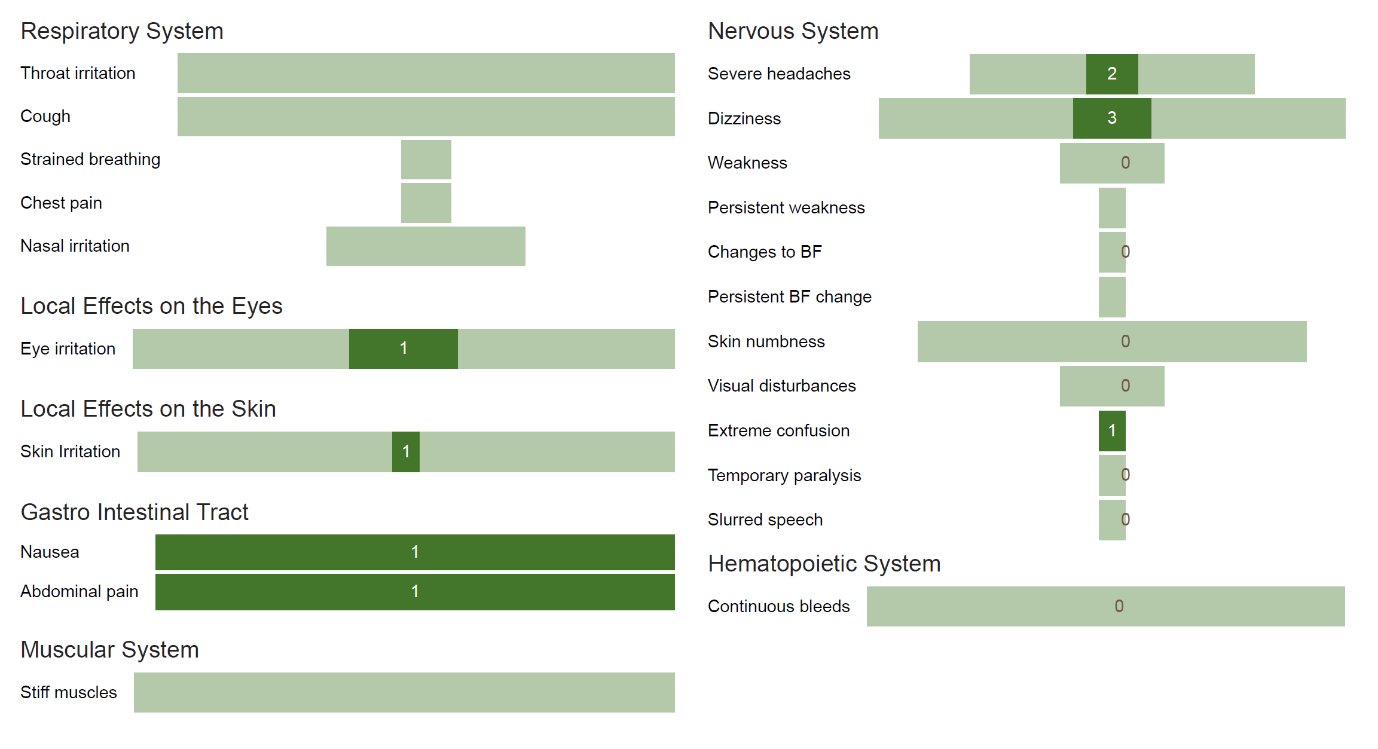


Supplementary Figure 7: The frequency of all symptoms reported following reports of UAPP by profenofos in Jamaica (n=6). Key: **Eye irit.** relates to eye irritation; **eyelid sw.** relates to eyelid swelling; **ab. Pain** relates to abdominal pain; **chest pain** related to a sharp stabbing pain in your chest which feels worse when you cough; **Weakness** includes any of the following symptoms: slowness or weakness when carrying out routine tasks, difficulty in walking or with balance, tremors or shaking, abnormal involuntary posture, abnormal movements of the tongue, jaw, face, arms, legs, neck or trunk; **Changes to BF** includes any of the following symptoms: increased or decreased salivation, decreased sweating, difficulty in urinating, constipation; **Persistent** relates to symptoms present for 48 hours or more.


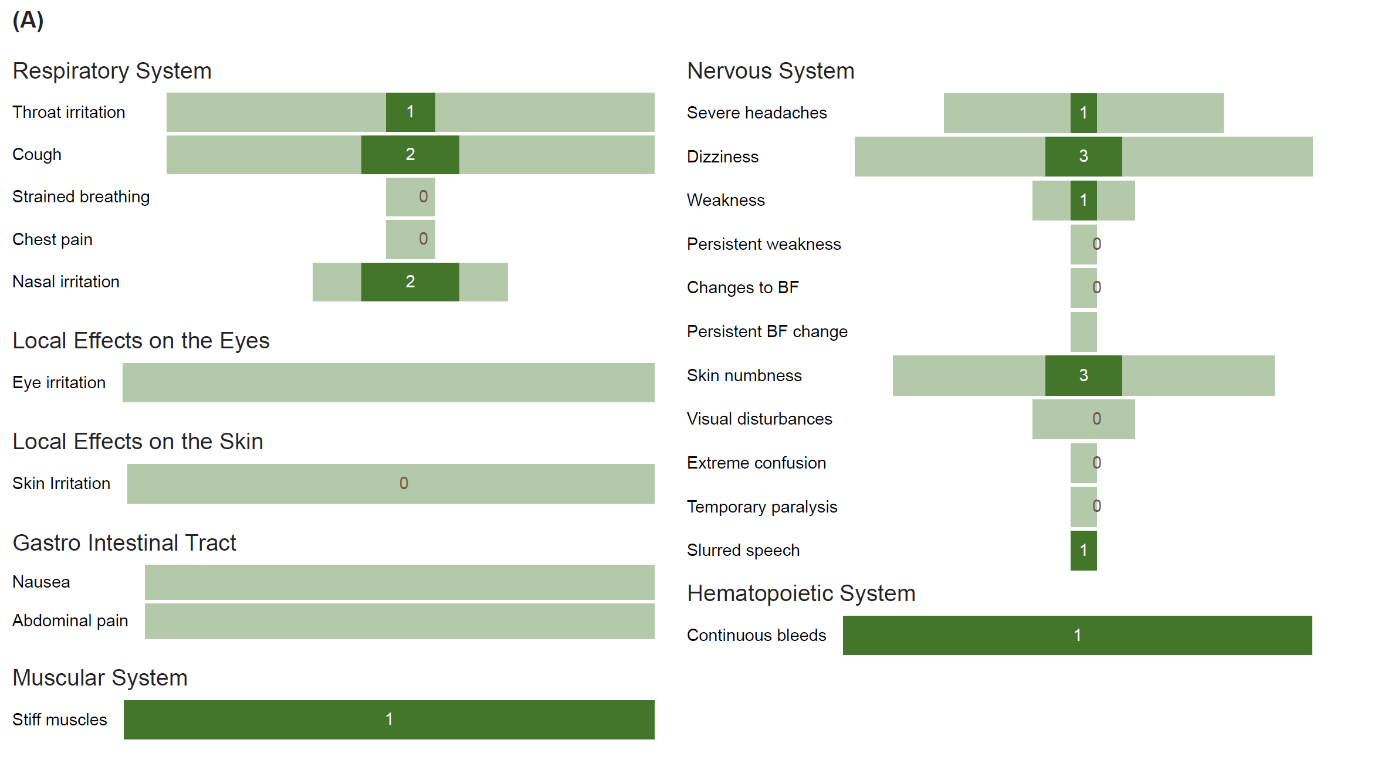


## Supplementary Tables

Supplementary Table 1: Pesticide trade names reported across both surveys in Jamaica and Trinidad, associated active ingredients and their WHO hazard classification.

| **Pesticide Trade Name** | **Active Ingredient** | **WHO Hazard classification** | **Country Reported In** |
| --- | --- | --- | --- |
| Agrinate-90 | Methomyl (90%) | Ib | Trinidad |
| Basta | Glufosinate-ammonium (200 g/L) | II | Trinidad |
| Belis | Boscalid (25.2%) + pyraclostrobin (12.8%) | U + n/a | Trinidad |
| Binder | Fluroxypr (200 g/L) | U | Trinidad |
| Caprid 20 SL | Acetamiprid (20%) | II | Jamaica and Trinidad |
| Caratrax 5EC | Lambda-cyhalothrin (5%) | II | Jamaica and Trinidad |
| Commando | Imidacloprid (35%) | II | Trinidad |
| Cure | Abamectin (1.8%) | n/a | Jamaica and Trinidad |
| Cypro | Profenofos (40%) + Cypermethrin (4%) | II + II | Trinidad |
| Diazinon | Diazinon (48%) | II | Jamaica and Trinidad |
| Dithane | Mancozeb (37%) | U | Jamaica |
| Fastac 5EC | Alpha cypermethrin (5.0%) | II | Trinidad and Trinidad |
| Gramoxone | Paraquat (200 g/L) | II | Jamaica and Trinidad |
| Gramoxone 200SL | Paraquat (200 g/L) | II | Jamaica |
| Gramoxone Super | Paraquat (200 g/L) | II | Trinidad |
| Indox-15SC | Indoxacarb (15%) | II | Trinidad |
| Karate 5EC | Lambda-cyhalothrin (5%) | II | Jamaica and Trinidad |
| Karate Zeon 5 CS | Lambda-cyhalothrin (50 g/L) | II | Jamaica and Trinidad |
| Lannate | Methomyl (29%) | Ib | Trinidad |
| Malathion | Malathion (57%) | III | Trinidad |
| Mancozeb | Mancozeb | U | Jamaica |
| Methomyl | Methomyl | Ib | Trinidad |
| Mocap | Ethoprophos (10%) | Ia | Trinidad |
| Pillar | Fenoxaprop-P-Ethyl (9.3%) | III | Trinidad |
| Pirate | Chlorfenapyr (360 g/L) | II | Trinidad |
| Rapid 5EW | Lambda-cyhalothrin | II | Trinidad |
| Regent | Fipronil (5%) | II | Trinidad |
| Rogor | Dimethoate (30%) | II | Trinidad |
| Roundup | Glyphosate | III | Trinidad |
| Selecron 500EC | Profenofos (500 g/L) | II | Jamaica |
| Silvacur Combi EC | Tebuconazole + Triadimenol/ 300 (225+75 g/L) | II + II | Trinidad |
| Supertak-10EC | Alpha cypermethrin (10%) | II | Trinidad |
| Swiper Herbicide 480+ | Glyphosate isopropylamine | III | Trinidad |
| Thiovin-275SC | Thiodicarb | II | Trinidad |
